# Enhanced Degradation of Antibiotic by Peroxydisulfate Catalysis with CuO@CNT: Simultaneous ^1^O_2_ Oxidation and Electron-Transfer Regime

**DOI:** 10.3390/molecules27207064

**Published:** 2022-10-19

**Authors:** Jia Liu, Chao Ding, Sicheng Gong, Kun Fu, Huiping Deng, Jun Shi

**Affiliations:** Key Laboratory of Yangtze River Water Environment Ministry of Education, Shanghai Institute of Pollution Control and Ecological Security, College of Environmental Science and Engineering, Tongji University, Shanghai 200092, China

**Keywords:** peroxydisulfate, nonradical process, singlet oxygen, trivalent copper, electron transfer

## Abstract

The nonradical process in the peroxydisulfate (PDS) oxidation system is a promising method for antibiotic removal in water. In this study, CuO@CNT was successfully synthesized by a facile approach to catalyze PDS. The removal efficiency of the antibiotic sulfamethoxazole (SMX) was 90.6% in 50 min, and the stoichiometric efficiency (ΔSMX/ΔPDS) was 0.402. The very different degradation efficiency of common organic contaminants revealed the selective oxidation of the surveyed system. The process of ^1^O_2_ oxidation and the electron-transfer regime was exhibited by chemical quenching tests, electron paramagnetic resonance (EPR) determination, a UV–vis spectrophotometer, X-ray photoelectron spectroscopy (XPS) detection, and cyclic voltammetry (CV) measurements. Sustainable catalysis was promoted by the circulation between the surface electron-rich centers of Cu(II) and Cu(III). Dissolved oxygen (DO) and a metastable Cu(III) intermediate contributed to the generation of ^1^O_2_. Still, a portion of SMX was removed by the mildly activated PDS. Moreover, the influence factors (pH, dosage, water matrix) were examined, and suppressions were acceptable by common anions and real water. Distinguished from the radical process, unique intermediate products were ascertained via the theoretical calculation and liquid chromatography–mass spectrometry (LC-MS) detection. Furthermore, CuO@CNT showed a satisfactory activation ability in the cycling experiments. Overall, this study developed CNT to be a supporter of CuO, unveiled the mechanism of catalysis, and evaluated the application potential of the nonradical process.

## 1. Introduction

Nonradical processes in peroxydisulfate (PDS) and peroxymonosulfate (PMS) have been increasingly studied [1,2,3]. They are dominated by singlet oxygen (^1^O_2_) [4,5], metastable-activated PS (PDS or PMS) bonded on the surface of catalysts [6,7], and high valence metal [8,9], instead of sulfate radical (SO_4_^•−^) and hydroxyl radical (•OH) in traditional advanced oxidation processes (AOPs). The oxidative species contribute to the selective removal of contaminants because of their mild redox potential [10,11]. The latest publications have expounded convincing superiorities, such as high PS utilization efficiency and resistance to complex water matrices [12,13,14]. Heterogeneous catalytic PS oxidation is the major contributor to the nonradical process, and the catalysts involved can be generally distinguished into carbon-based and metal-based materials [15]. Metal-based catalysts usually include the oxides and hydroxides of cobalt [16], manganese [17], iron [18], copper [19], and nickel [20]. Researchers have also developed complexes with particular morphology and dimensionality, such as metal-organic frameworks (MOFs) [21] which are coordination compounds extending through repeating coordination entities in two or three dimensions with potential voids, and transition-metal carbides/nitrides (MXene) [22] which are two-dimensional layered materials. Among them, copper oxide (CuO) is one of the first catalysts used for the nonradical process. In 2014, Zhang et al. [6] proposed the direct electron abstraction from 2,4-dichlorophenol (2,4-DCP) to PDS relying on an outer-sphere complexation by CuO, which was obtained by the calcination of solid copper nitrate. SO_4_^•−^ was not generated in the system, and the same rate of the PDS consumption and the 2,4-DCP degradation demonstrated unexceptionable application prospects. Based on an analogous procedure, Du et al. [23] also obtained CuO nanoparticles and found that p-chloroaniline was mostly degraded by the activated PDS, and slightly by SO_4_^•−^ and •OH. Cu(II) was the active site in the PDS’s direct oxidation process, whereas Cu(II) and Cu(III) were cycled in the radical process. In another regime [24], ^1^O_2_ was generated by a metastable copper intermediate. Cu(III) not only participates in the redox cycle of Cu(I, II, and III), but can also directly oxidize certain contaminants [25,26]. Recently, researchers have established reaction equations as shown in Equations (1)–(5) [27]; Equation (1) occurs at an extremely slow rate and Equation (3) is thermodynamically unfavorable [25].

(1) $${\mathrm{Cu}(\mathrm{II})+S}_{2}O_{8}^{2-}\;\to {\;\mathrm{Cu}(\mathrm{III})+\mathrm{SO}}_{4}^{\text{•}-}{+\mathrm{SO}}_{4}^{2-}$$

(2) $${\mathrm{Cu}(I)+S}_{2}O_{8}^{2-}\;\to {\;\mathrm{Cu}(\mathrm{II})+\mathrm{SO}}_{4}^{\text{•}-}{+\mathrm{SO}}_{4}^{2-}$$

(3) $${\mathrm{Cu}(\mathrm{II})+\mathrm{HSO}}_{5}^{-}\;\to {\;\mathrm{Cu}(I)+\mathrm{SO}}_{5}^{\text{•}-}{+H}^{+}\;$$

(4) $${\mathrm{Cu}(I)+\mathrm{HSO}}_{5}^{-}\;\to {\;\mathrm{Cu}(\mathrm{II})+\mathrm{SO}}_{4}^{\text{•}-}{+\mathrm{OH}}^{-}$$

(5) $${\mathrm{Cu}(I)+\mathrm{HSO}}_{5}^{-}\;\to {\;\mathrm{Cu}(\mathrm{III})+\mathrm{SO}}_{4}^{2-}{+\mathrm{OH}}^{-}$$

The activation ability of CuO and the variable valence states of Cu indicate a promising approach to activating PS [28]. Over the past decades, researchers have employed a combination of materials as a novel strategy to enhance metal oxides [29]. Carbon nanotubes (CNTs), a typical nanocarbon material, are composed of one or more rolled-up graphene layers with hexagonal arrays [30]. With a large surface, and mechanical and thermal stability, CNTs exhibit outstanding electron-transfer capability [31], resulting in a wide application in catalysis [32,33]. As for the PS oxidation systems, the CNT-mediated electron-transfer regime has been verified by Yun et al. [34] and Duan and Wang’s group [11]. Furthermore, CNTs are a promising platform to develop novel catalytic composites which have showed an exceptionally good performance in catalytic processes, such as chemical catalysis, electrocatalysis, and photocatalysis [35]. Thus, in this study, we designed a simple and cost-effective approach to modify CNTs with CuO. Due to the costs of research and process development, single-wall CNT prices are orders of magnitudes higher than for multi-wall CNTs [36]. Thus, a multi-wall CNT was used instead of a single-wall CNT.

The activation ability may be predictable and convincing because of the following reasons: (i) CNTs promoted the dispersion of CuO, further increasing the number of active sites; and (ii) the electron transfer from the carbon material to the metal [37] may facilitate the activation. Moreover, ion leaching may be decreased by employing the supporter. Practical applications of catalytic composites are promising, whereas academics estimate that the processes have not been fully realized and many knowledge gaps need to be bridged [35]. Thus, this work emphasized the mechanism process and a practical study was conducted to reveal the application potential of CuO in the nonradical process.

Sulfamethoxazole (SMX) is one of the earliest and most broadly used antibiotics [38]. However, the unabsorbed SMX is unaltered and excreted into sewage systems, leading to toxic effects on aquatic ecosystems and organisms. SMX concentration reaches 2.0 µgL^−1^ in surface water and final sewage effluents in some countries [39]. Moreover, antibiotic resistance is a global health crisis attributed to unrestricted use in humans and animals [40]. Various water treatment technologies are employed to remove antibiotics in water, such as chemical oxidation, physical adsorption, biodegradation, and photocatalysis [41]. In this study, we used SMX as the model antibiotic for mechanism exploration and application analysis. This work aims to develop a facile synthesized catalyst for the nonradical process in the PS process. For the first time, the combination of two activators that induce nonradical processes in heterogeneous PS systems was reported. ^1^O_2_, in situ Cu(III), and surface-active PS were involved in this distinct mechanism, which contributed to catalysis rather than activation. The cycle of divalent and trivalent copper was achieved, and the contribution of Cu(III) was investigated by a variety of strategies. In the study, four aspects were performed: (i) the degradation of SMX and other model organics, and the utilization of PS; (ii) the mechanism of the catalytic process; (ⅲ) the effects of dosage and the water matrix; and (ⅳ) the possible transformation pathway of SMX.

## 2. Results and Discussion

### 2.1. Characterization

**Figure 1:**
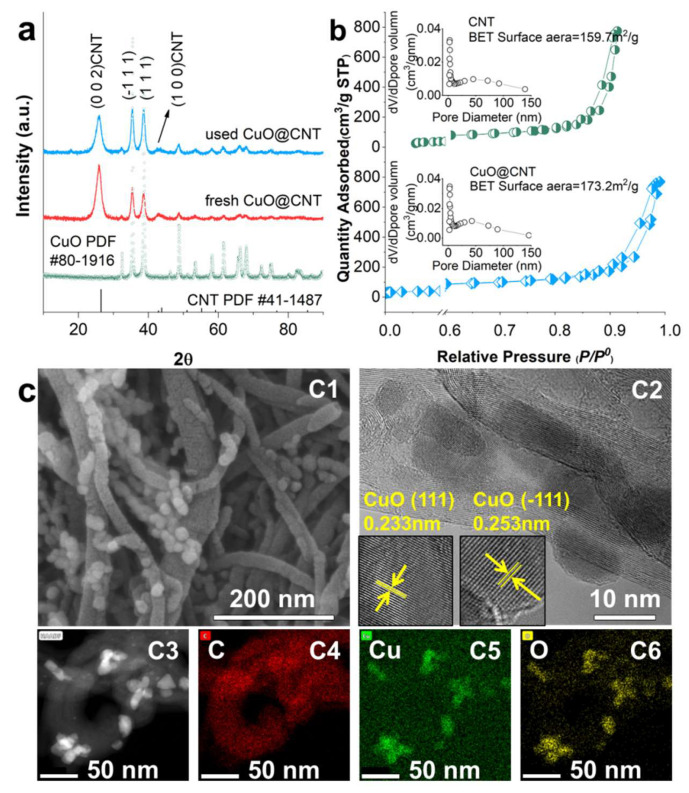
(**a**) XRD patterns of CuO and CuO@CNT; (**b**) BET measurements for CNT and CuO@CNT; (**c1**) SEM images; (**c2**) HRTEM images; (**c3**–**c6**) elemental mapping data of CuO@CNT.

**Figure 2:**
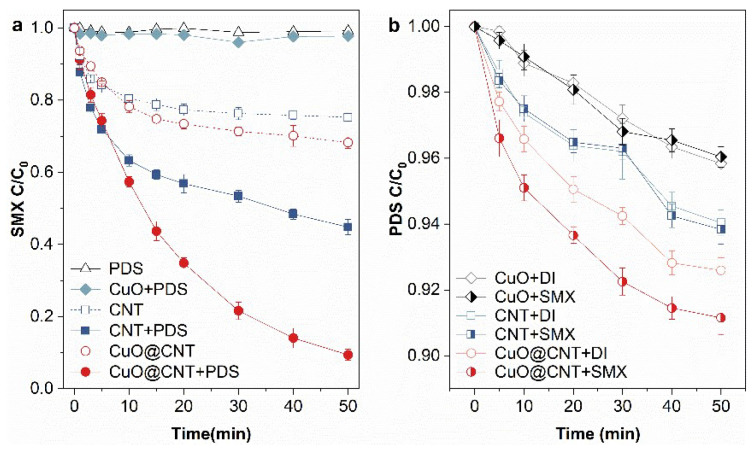
(**a**) SMX adsorption and degradation; (**b**) PDS adsorption and decomposition by CuO, CNT, and CuO@CNT. Conditions: SMX = 39.5 µM, PDS = 1.0 mM, Activator = 0.1 g/L, pH = 5.6 ± 0.1.

X-ray diffraction (XRD) patterns of the synthesized materials are shown in Figure 1a. The results indicate the CuO peaks (PDF no. 80-1916) are well fitted. The diffraction crystal planes of the CNTs (PDF no. 41-1487) are also displayed. The SEM and TEM images are presented in Figure 1c. As shown in Figure 2(c1), CuO particles bond to the ektexine of CNTs. The lattice spacing of 0.233nm corresponds to the (1 1 1) plane, whereas the lattice spacing of 0.253 nm corresponds to the (−1 1 1) plane, as shown in Figure 2(c2). As indicated in Figure 2(c3–c6), CuO particles are well distributed. The characterizations above show that CuO@CNT was successfully formed. XRD patterns of samples with different copper loading contents are shown in Supplementary Materials Figure S1. Moreover, the loaded CuO does not destroy the CNT structure, but increases the surface area, as demonstrated by the Brunauer–Emmet–Teller (BET) characterization results (Figure 1b). The N_2_ adsorption/desorption isotherms match with the type II isotherm, and the type Ⅳ hysteresis loop demonstrates the presence of a mesopore structure, according to the IUPAC classification. However, CuO exhibits a small surface area and the mesopore structure is not distinct (shown in Figure S2). The compositions of the materials are also verified by FTIR spectroscopy, and the results are shown in Figure S3. CNTs and CuO@CNT exhibit a band at 1580 cm^−1^, which is attributed to the C=C cyclic alkene vibration. The obvious band at 522 cm^−1^ is relative to the Cu-O vibration of CuO [42]. Notably, the Cu-O vibration of CuO@CNT is relatively weak due to the low copper content.

To check the loading content of copper and the mass balance, a digestion experiment was carried out. CuO@CNT was dispersed in a nitric acid solution and then boiled on a heating plate for 2 h. With the copper loading content increased from 15% (wt%, of CuO@CNT) to 35%, the SMX degradation was first increased and then decreased (Figure S4). With a lower loading amount, CuO was well distributed on the CNT, the mesopore structure was intact, the specific surface area was maintained at a high level, and the catalytic activity mainly depended on the number of active sites. Thus, the performance of 25% was better than that of 15%. On the other hand, when the loading content was more than 25%, the crystallite size of CuO may be increased, and the agglomerated CuO lowered the specific surface area and damaged the mesopore structure [43], which may be responsible for the weakening of the catalytic activity. The optimum CuO@CNT was singled out and the copper content was 25%.

### 2.2. Degradation of SMX and Consumption of PDS

The SMX degradation efficiency of different oxidation systems was inspected. Under the conditions of 39.5 µM SMX, 0.1g/L CuO@CNT, and 1 mM oxidant, the degradation efficiencies were 100%, 90.6%, and 25.2% in 50 min by PMS, PDS, and H_2_O_2_, respectively (Figure S5). Although the highest value was obtained in the PMS system, the ion leaching of Cu^2+^ (7.852 mg/L) was unacceptable, far above the Integrated Wastewater Discharge Standard (2 mg/L) and Environmental Quality Standards for Surface Water (1 mg/L) of China. In the PDS system, ion leaching was decreased to 0.497 mg/L. In terms of the activation ability, the ion leaching, and the reagent cost (PDS, USD 0.74 /kg; PMS, USD 2.2 /kg; H_2_O_2_, USD 1.5 /kg) [6], PDS was employed. Because of the asymmetrical structure, PMS was more easily activated [44]. At the same time, metal leaching increased dramatically because of the more effective activation and the more acidic solution. When PMS was employed, the pH value decreased to 3.4 at the end of the reaction (4.5 in the PDS system).

Specific SMX and PDS concentration variations are shown in Figure 2. In Figure 2a, it can be found that PDS alone had a negligible effect on SMX degradation. In the CuO/PDS system, SMX degradation cannot be detected, indicating any generation of reactive oxygen species (ROS) or direct oxidation. CNTs adsorbed 25.8% SMX in 50 min. This result is analogous to the previous publications [45,46], in which the aromatic organics interact with CNTs by hydrophobic driving forces, electrostatic interactions, π–π interactions, and hydrogen bonds. As for the activation ability of CNTs, 55.2% of SMX was removed in 50 min. However, CuO@CNT improved the degradation efficiency to 90.6%, which was much higher than the sum of the CuO and the CNT systems. The results show that the combination of CuO and CNTs effectively enhanced the activation ability for PDS. The difference (ΔSMX) between degradation efficiency and adsorption efficiency is 58.8%, indicating that CuO@CNT acts as an effective activator rather than an adsorbent. Validation was conducted (Figure S6) to verify the activation by Cu^2+^. No activation ability was exhibited by Cu^2+^ of 25 mg/L (which was equivalent to the cooper content in 0.1 g/L CuO@CNT). With CNTs, the SMX degradation increased from 55.2% to 59.3% with the introduction of Cu^2+^. Cu^2+^ was introduced in the form of CuCl_2_; thus, the same amount of Cl^−^ (NaCl) was examined, but no influence was found. To summarize, the activation ability of CuO@CNT relies on heterogeneous reactions. As shown in Figure 1b, the BET surface areas of CuO@CNT and CNTs are 173.2 m^2^/g and 159.7 m^2^/g, respectively. Thus, the overall adsorption efficiency of CuO@CNT was higher because of the bigger surface area (shown in Figure 2a). Nevertheless, CuO may initiate aggregation [43] of CNTs, and thus, at the start of the reactions, the adsorption efficiency of SMX was relatively low by CuO@CNT. In the mid- and late-reaction, the superior was shown. Additionally, in the oxidation process, CuO@CNT showed an advantage at 10 min, and the removal efficiency of SMX by CuO@CNT and CNTs are 42.7% and 25.7%, respectively. In the beginning, the performance of CuO@CNT was poorer than that of CNTs. That indicates that SMX was rapidly adsorbed and the surface reaction may dominate the oxidation in 10 min.

As for the consumption of PDS (shown in Figure 2b), CuO consumed 42.7 µM and 40.1 µM PDS in deionized water and the SMX solution, respectively. PDS congregated at the surface of CuO, whereas it was not activated for SMX removal. CNTs consumed 59.6 µM and 61.6 µM PDS in deionized water and the SMX solution, respectively. It can be seen that the addition of SMX did not cause an obvious variation in the consumption of PDS by CuO or CNTs. As for CuO@CNT, 74.0 µM PDS was consumed in deionized water, which means more active sites can be occupied by PDS on the surface of CuO@CNT than that on CNTs (or CuO). This result may be attributed to the superior dispersion of CuO particles attached to CNTs. With SMX, the PDS consumption by CuO@CNT increased to 88.5 µM, indicating that SMX may act as an inducer for PDS decomposition. By our calculation, the stoichiometric efficiency (ΔSMX/ΔPDS) is 0.402 using CuO@CNT as an activator. Researchers have reported values of 0.188, 0.052, and 0.118 in heterogeneous catalytic PDS oxidation systems for SMX degradation using CNTs [47], micrometric Fe^0^ [48], and trimetallic metal (AgCoFe) [49] as activators, respectively. Among these, CuO@CNT better utilized PDS. Based on the consumption of PDS and the degradation of SMX, CuO@CNT can be considered to be a good activator for PDS to degrade antibiotics, and SMX-accelerated PDS decomposition may imply a complex mechanism.

### 2.3. Mechanism Study

#### 2.3.1. Selective Oxidation

The degradation of other model contaminants was further examined. The removal efficiency is depicted in Figure S7. As shown in Figure S8, pseudo-first-order kinetic fitting results of nitrobenzene (NB), p-chlorobenzene acid (*p*-CBA), furfuryl alcohol (FFA), ofloxacin (OFX), SMX, and 2,4-dichlorophenol (2,4-DCP) degradation illustrate the selective oxidation ability of the CuO@CNT/PDS system. The apparent rate constants (k_obs_) of 0.0011, 0.0015, 0.0066, 0.027, 0.048, and 0.093 min^−1^ corresponded to NB, *p*-CBA, FFA, OFX, SMX, and 2,4-DCP degradation, respectively. As the chemical probes of •OH [50] and SO_4_^•−^ [51], NB and *p*-CBA can hardly be removed; thus, it can be predicted that the radicals were not significantly generated. FFA is the chemical probe or quencher for ^1^O_2_ because of the high order of the second-order rate constant (~10^8^ M^−1^s^−1^) [52]. It is worth noting that other ROS such as radicals can also quickly consume FFA (~10^10^ M^−1^s^−1^) [53]. Here, 31% of FFA was degraded, indicating that ^1^O_2_ and/or radicals were involved. Antibiotic SMX and OFX can be almost eliminated in 50 min, and effective ROS species may be generated in the surveyed system. 2,4-DCP can be completely degraded in 20 min. 2,4-DCP, which is the earliest model pollutant in PS oxidation studies [53], has been proved to be oxidized by radicals [54], ^1^O_2_ [5], and the activated PDS–catalyst complex [6]. The selective oxidation phenomenon of this surveyed system reveals that the key oxidative species may not be •OH or SO_4_^•−^.

#### 2.3.2. Quenching and EPR Tests

**Figure 3:**
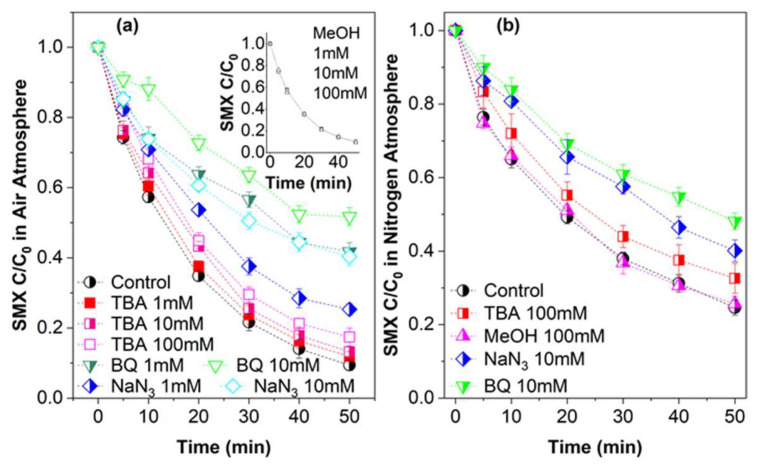
Chemical quenching in SMX degradation by CuO@CNT/PDS (**a**) in air; (**b**) in nitrogen. Conditions: SMX = 39.5 µM, PDS = 1.0 mM, Activator = 0.1 g/L, pH = 5.6 ± 0.1.

**Figure 4:**
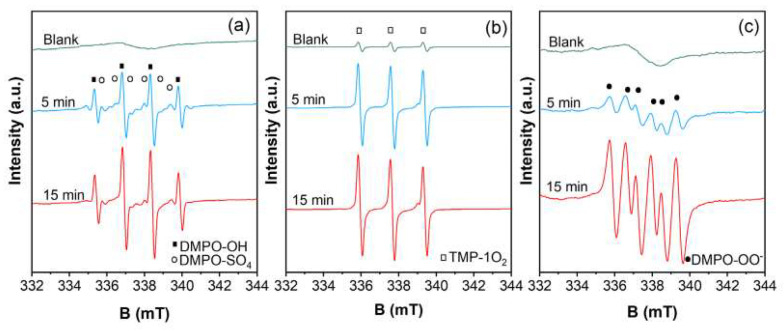
EPR spectra of CuO@CNT/PDS: (**a**) •OH and SO_4_^•−^, (**b**) ^1^O_2_, (**c**) O_2_^•−^. Conditions: PDS = 1.0 mM, CuO@CNT = 0.1 g/L.

*Tert*-butyl alcohol (TBA), methyl alcohol (MeOH), sodium azide (NaN_3_), and benzoquinone (BQ) were used as chemical quenchers since they have high reaction rate constants with ROS; details can be found in the Supplementary Materials (Table S1). The inhibitions of SMX degradation are shown in Figure 3a. MeOH and TBA are used to quench •OH and SO_4_^•−^. Effects made by MeOH are shown in the inset of Figure 3a, where no inhibition can be found at any concentration. There are two conjectures that can be given: (i) no radical was generated; and (ii) radicals were generated but did not contribute to the degradation. To further reveal the internal situation, an EPR measurement was carried out to check for the existence of •OH and SO_4_^•−^. As shown in Figure 4a, the typical additive product signals of DMPO and •OH/SO_4_^•−^ were found. For a prudential reason, TBA or MeOH was injected at a certain moment of the reaction, and the strong signal disappeared, as shown in Figure S9. A conclusion can be drawn that radicals were not the dominant oxidative species in the surveyed system. The finding is in accordance with the earlier study [43], which is a conclusion that cannot be made only according to quenching tests, and the results of EPR should also be taken into account.

Attention was paid to the limited suppression by TBA (shown in Figure 3a), as the SMX degradation efficiency decreased by 2.5%, 3.8%, and 8.0% in the presence of 1 mM, 10 mM, and 100 mM TBA, respectively. The different inhibitions by MeOH and TBA may be caused by some reactive species which are more likely to be captured by TBA rather than MeOH, and directly or indirectly contributed to SMX degradation. Comparatively speaking, MeOH is identified to be hydrophilic [55] and TBA is hydrophobic [56]. TBA was apt to capture surface reactive species. Cu(III) has been found in the PS catalytic oxidation system [27], which can react with TBA at the rate of k = (1 ± 0.4) × 10^7^ M^−1^s^−1^ [57]. It is reasonable to infer the existence of ≡Cu(III)/Cu(III) according to the quenching effect of TBA.

In some published research, ^1^O_2_ and $O_{2}^{\text{•}-}$ were verified to be reactive species in the heterogeneous PS catalytic systems. NaN_3_ was used to capture ^1^O_2_ [4,58]. The degradation efficiency of SMX declined by 15.9% and 30.9% in the presence of 1 mM and 10 mM NaN_3_, indicating the existence of ^1^O_2_. Previous publications claimed that BQ could accelerate activation [59,60]. For prudential reasons, the SMX concentration was examined in the BQ/PDS/SMX system (BQ = 1 mM), whereas no such conclusion can be made in this work. In the CuO@CNT/PDS system, 1 mM BQ greatly inhibited the degradation of SMX, as only 58.2% of SMX was degraded. When the concentration of BQ rose to 10 mM, 48.2% SMX was degraded. BQ is used to quench $O_{2}^{\text{•}-}$ [61], which is the precursor of ^1^O_2_. Researchers [1] suggested that multi-strategies should be used for the verification of the occurrence of ^1^O_2_, and thus, EPR measurements were carried out for further determination of ^1^O_2_ and, also, $O_{2}^{\text{•}-}$. The results are shown in Figure 4b,c, where the typical 1:1:1 three-line spectrum displayed the adduct of TMP and ^1^O_2_ [62], and the spectrum of the DMPO-O_2_^•−^ spin adduct [62] is distinct. Overall, the existence of ROS and their contribution are confirmed by chemical quenching and EPR measurements.

The effects of dissolved oxygen (DO) in the PS oxidation system have been recognized [63,64]. DO can convert to $O_{2}^{\text{•}-}$ by accepting an electron [65]. Therefore, tests were carried out without DO for further study. N_2_ was purged into the solution to expel DO, and then, the tests were conducted in an N_2_ atmosphere. The results are shown in Figure 3b. The SMX degradation efficiency declined to 75.4%, as mentioned before, and 90.6% of SMX was degraded in the air condition; therefore, 15.2% degradation efficiency was attributed to DO. An impressive positive effect was contributed by DO in this way: once it accepts an electron, DO converts to $O_{2}^{\text{•}-}$, which is a crucial source of ^1^O_2_ (Equation (6)) [66]. Still, MeOH had no impact on SMX degradation in the N_2_ atmosphere. As shown, the difference in the suppression by NaN_3_ was 15.4%, which represented the contribution of ^1^O_2_ derived from DO. It is numerically analogous to the difference between the control tests (15.2%). As for BQ, the difference in suppression was 18.9%, which represented the contribution of $O_{2}^{\text{•}-}$ derived from DO. As for TBA, the difference was 3.20%, implying the generation of ≡Cu(III) was relatively less influenced. Thus, DO contributed to about 15% of the SMX degradation by generating ^1^O_2_. Thus, the participation of ^1^O_2_ and ≡Cu(III) in SMX degradation is verified, as was DO’s contribution to the generation of ^1^O_2_.

O_2_^• ─^ + H_2_O → ^1^O_2_ + H_2_O_2_ + OH^−^ (6)

#### 2.3.3. Surface Trivalent Copper

**Figure 5:**
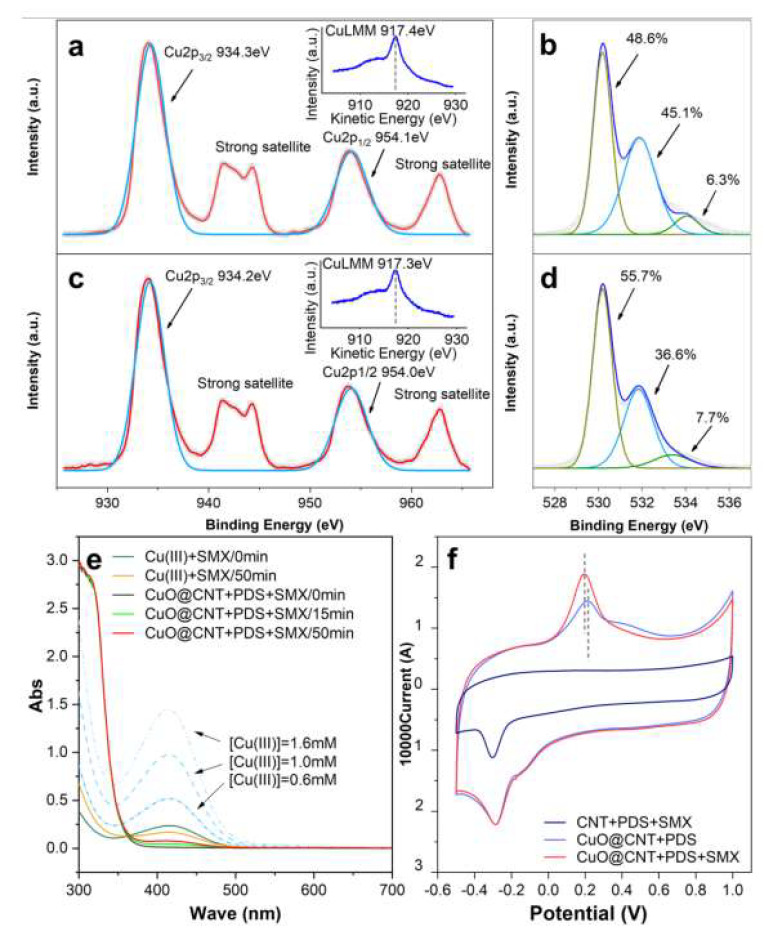
XPS of (**a**) Cu2p of fresh CuO@CNT, (**b**) O1s of fresh CuO@CNT, (**c**) Cu2p of used CuO@CNT, (**d**) O1s of used CuO@CNT, (**e**) UV–vis spectrum of Cu(III), (**f**) cyclic voltammograms (CVs) on CNTs and CuO@CNT electrode in 0.5 M Na_2_SO_4_ and 1.0 mM PDS; scan rate = 50 mVs^−1^. Conditions: PDS = 1.0 mM, CuO@CNT = 0.1 g/L, SMX = 39.5 µM, Cu(III) = 0.4 mM, (**e**) pH = 13.0 ± 0.1, (**f**) pH = 5.6 ± 0.1.

As indicated in Figure 5a,c, satellite and auger spectrums indicate only Cu(II) in CuO@CNT before and after the reaction, as Cu(I) cannot be found in the characterization tests. The binding energies at approximately 934.3 and 954.1 eV correspond to the Cu 2p3/2 and Cu 2p1/2 peaks, respectively [67]. The strong satellite peaks at around 942.0 and 962.9 eV correspond to Cu(II). Moreover, the LMM Auger peak at around 917.4 eV also points to Cu(II). Although indicated in chemical quenching tests, Cu(III) is instable and cannot be detected directly [68]. By comparison, the Cu(III) periodate complex is easy to be synthesized and detected [69], and thus, diperiodato-cuprate(III) ([Cu(HIO_6_)_2_]^5−^) was prepared. Absorbance at about a 415 nm wavelength by UV–vis spectra is shown in Figure 5e, indicating Cu(III) was successfully synthesized and steadily existed [25]. In the surveyed system, potassium periodate (KIO_4_) was added and the solution’s pH was adjusted to 13 to complex the possible Cu(III) and make the complex stable. As shown, the absorbance peak at 415 nm can be observed with the increasing reaction time. It can be inferred in the surveyed system that Cu(III) was generated.

The evolution of Cu(III) and oxygen may be the key point to elucidating the reaction mechanism. Oxygen plays a critical role in the catalytic oxidation processes of transition metal oxides [70]. Before and after employing, the XPS characterization of CuO@CNT was carried out to inspect the change in oxygen species. The subtle change in O1s binding energy is illustrated in Figure 5b,d. The peak located at 530.2 eV indicates lattice oxygen (O^2−^), whereas the peak at 531.8 eV is ascribed to adsorbed oxygen; additionally, the peak at 533.4 eV is assigned to surface hydroxyl species [71]. It can be seen that for the used CuO@CNT, the proportion of O^2−^ increased from 48.6% to 55.7%, adsorbed oxygen decreased from 45.1% to 36.6%, and surface hydroxyl species slightly increased from 6.3% to 7.7%. The changes mainly lie in the proportion of lattice and adsorbed oxygen. The transformation of adsorbed oxygen species can be described by Equation (7) [70]. The ionic form is generated by accepting electrons, and finally, O^2−^ is formed.

CuO@CNT is a composite activator, and the generation of Cu(III) was probably a benefit of the CNTs. CuO is a *p-*type semiconductor [72], and for CuO@CNT, where electron transfers from the carbon material to the metal [73], this means that Cu(II) is the electron-rich center. This process undoubtedly enhances the possibility of Cu(II) as the reactive site [74,75], and ≡Cu(III) can be formed to accomplish charge compensation [62]. Therefore, along with the transformation of oxygen species, ≡Cu(III) was generated [69]. ≡Cu(III) can react with surface PDS, causing a metastable copper intermediate to form as in Equation (8), and then, $O_{2}^{\text{•}-}$ was generated, as in Equation (9) [24]. $O_{2}^{\text{•}-}$ was recognized as the precursor of ^1^O_2_, as depicted in Equation (6). When O^2−^ was dislodged, oxygen vacancy was left on the surface of metal oxides, and the metal valence changed accordingly [76]. In summary, the reactive site Cu(II) is the electron-rich center attributed by CNTs providing electrons. ≡Cu(III) was generated by the transformation of oxygen species. ^1^O_2_ was generated by the precursor $O_{2}^{\text{•}-}$, which comes from: (i) DO and (ii) adsorbed oxygen.

(7) $$O_{2\left(\mathrm{ads}\right)}\;\to {\;O}_{2(\mathrm{ads})}^{-}\;\to {\;O}_{\left(\mathrm{ads}\right)}^{-}\;\to {\;O}_{\left(\mathrm{lattice}\right)}^{2-}$$

(8) $$\equiv {\mathrm{Cu}}^{\mathrm{III}}{-\mathrm{OH}+S}_{2}O_{8}^{2-}\;\to \;\equiv {\mathrm{Cu}}^{\mathrm{III}}{-O-O-\mathrm{SO}}_{3}$$

(9) $$\equiv {\mathrm{Cu}}^{\mathrm{III}}{-O-O-\mathrm{SO}}_{3}{+S}_{2}O_{8}^{2-}{+H}_{2}O\;\to \;\equiv {\mathrm{Cu}}^{\mathrm{II}}{-\mathrm{OH}+O}_{2}^{\text{•}-}{+\mathrm{SO}}_{4}^{2-}{+H}^{+}$$

In the meantime, the possibility of SMX degradation by Cu(III) cannot be ignored [26]; hence, a series of validation experiments were carried out. [Cu(HIO_6_)_2_]^5−^ was used to represent Cu(III), as mentioned above. In the SMX/ [Cu(HIO_6_)_2_]^5−^ system, as depicted in Figure S10, SMX degradation was 26.30% by Cu(III), and the [Cu(HIO_6_)_2_]^5−^ dosage was in accordance with the Cu loading content of CuO@CNT. The variation in the Cu(III) absorbance was observed synchronously, as shown in Figure 5e, and the peak at 415 nm was flattened out at the end of the reaction, indicating the concentration of Cu(III) also declined after the reaction. When PDS was added, no significant improvement could be found, and the PDS concentration was stable, as shown in Figure S10. It may be because Cu(III) of [Cu(HIO_6_)_2_]^5−^ was completely complexed, and thus, PDS can hardly participate in the complexation reaction as in Equation (8). In the surveyed system, for simulating the homogeneous system, the pH was adjusted to 13 and the SMX degradation was 52.98%. In parallel experiments, KIO_4_ was added to the complex Cu(III) to make Cu(III) have a longer lifetime. With the increasing reaction time, the absorbance at about 415 nm can be observed (shown in Figure 5e). At 50 min, the peak was more pronounced than at 15 min, indicating the accumulation of Cu(III). However, in this reaction, the SMX degradation efficiency was slightly decreased to 50.74%, and Cu(III) with a longer lifetime did not promote the reaction. The loss of degradation efficiency was due to the supersession of PDS complexation by KIO_4_, whereas the direct oxidation of SMX did not happen or the little oxidation could not recover the loss. It is reasonable to infer Cu(III) was more likely to participate in the reactions indirectly with PDS instead of oxidizing SMX directly in the surveyed system.

#### 2.3.4. Electron-Transfer Regime

^1^O_2_ has been verified to be prominent in the PS catalytic oxidation process, which can selectively oxidize organics including SMX [24]. In the standard state, the saturation value of DO is 9.08 mg/L, and PDS is 1 mM in standard tests. In this survey, the excessive dosage of the quencher NaN_3_ inhibited SMX degradation by 59.70%, meaning ^1^O_2_ caused the majority of the degradation. SMX degradation cannot be completely impeded by chemical quenchers in this work. As mentioned before, SMX accelerated the decomposition of PDS, and therefore, it is reasonable to propose a reaction between SMX and PDS. As described before, CuO selectively adsorbed PDS rather than SMX in the CuO/PDS/SMX system. The electron-rich center Cu(II) can weakly activate the adsorbed PDS [77] to degrade SMX directly, which means the electron-transfer regime occurs from SMX to PDS. This regime is supported by CV measurements (shown in Figure 5f). In contrast to the results of the system without SMX, the more obvious redox peak and the high current response are performed in the system with SMX. The phenomenon indicates a more intensive electron-transfer process [24] when PDS and SMX coexisted. In addition, the oxidation peak position shifts to a lower potential, implying that the reaction is more likely to occur [78]. The oxidation peak and reduction peak indicate reversible reactions [11]. CuO@CNT is likely to be a catalyst rather than an activator.

#### 2.3.5. Mechanism of the Nonradical Process and Reusability of Catalyst

All the experiments and analyses have been mentioned before confirming the nonradical process. PDS was efficiently utilized by CuO@CNT. ^1^O_2_ was the final oxidant of which the source was DO and the redox cycle was of ≡Cu(II) and ≡Cu(III). Moreover, a portion of SMX was directly oxidized by the activated PDS. Radicals were not the dominating oxides. There is reason to recognize the reaction as Equation (1) since SO_4_^•−^ was generated. •OH was generated through the hydrolysis of SO_4_^•−^. Though the process is slow, the leaching Cu^2+^ may activate PS at a relatively slow concentration; the early study published that the leaching Cu^2+^ at 0.4 mg/L slowly activated PDS and then generated SO_4_^2−^ and SO_4_^•−^, which was not vital in degradation [23].

Fresh and used CuO@CNTs were analyzed by XRD (Figure 1a). The crystal diffraction peaks of the used CuO@CNTs are still intact. For practical applications in water treatment, degradation tests with recycled CuO@CNT were carried out to explore its reusability (shown in Figure S11). CuO@CNT maintained a satisfactory catalytic performance in the first ternary cycles, and the SMX degradation efficiencies were 90.6%, 79.7%, and 72.5%. In the fourth cycle, only 61.3% of SMX was removed. Before the fifth cycle, the catalyst was filtrated and washed with ethyl alcohol, and then thermally treated at 400 °C. A total percentage of 82% of SMX was removed, attributing to the regeneration of active sites and the clearing of pores. The decrease is due to the reduction in the exposed crystal surface. The reusability indicates the possibility for practical use.

### 2.4. Effects

#### 2.4.1. pH

The effects of pH were examined. The pH_pzc_ of CuO@CNT is 5.1 (zeta potential is shown in Figure S12). SMX degradation efficiencies are shown in Figure S13a, and SMX distribution and catalyst charge are shown in Figure S13b. At the condition of pH = 5, the maximum degradation efficiency (94.7%) was attributed to the neutral surface of the catalyst, where both SMX and SMX^−^ can interact. When the pH was 3, the SMX concentration decreased rapidly in 10 min, whereas the relative severity of ion leaching under this condition restrained the reactions, and 82.8% SMX was removed in 50 min. When the pH was 5.6 or even higher (7, 9, 11), the negatively charged surface hindered the adsorption of SMX^−^, and thus, the degradation efficiency decreased. When pH was 11, the degradation efficiency was 62.1%; although alkaline activation has been proposed [79], it was not observed in this system.

Equation (10) occurs at alkaline conditions [68], whereas no obvious signal of Cu(I) in XPS characterization or acceleration in SMX degradation can be found at pH 7, 9, and 11, indicating such a reaction is unfeasible or slowly occurs in this work.

(10) $$\mathrm{Cu}\left(\mathrm{III}\right){+\mathrm{OH}}^{-}\;\to \;\mathrm{Cu}\left(I\right)+\text{•}\mathrm{OH}$$

#### 2.4.2. Dosage of SMX, CuO@CNT, and PDS

To deeply understand the effect of the dosage, kinetic fittings are illustrated in Figure S14. As shown, all the test results can be well fitted with a pseudo-first-order reaction model. The parameters can be found in Tables S2–S4. The SMX concentration had a negative effect on the degradation rate (Figure S14a), as k_obs_ changed from 0.0808 to 0.00713 as the dosage changed from 19.8 µM to 158 µM. As described in the inset figure, ln(k_obs_) vs. ln(C_SMX_) conforms to a linear rule, indicating that k_obs_ is proportional to a 0.441 power of the SMX concentration. Conversely, the CuO@CNT concentration had a positive effect (Figure S14b). By the same fitting calculation, k_obs_ is proportional to a 1.380 power of the catalyst concentration. The PDS concentration did not show such a regular impact (Figure S14c). Although the highest concentration of 4 mM resulted in the highest rate, no significant change can be found in the tests.

#### 2.4.3. Water Matrices

Traditional sulfate radical-based advanced oxidation processes (SR-AOPs) are highly susceptible to common anions in water due to the oxidation by radicals [1]. Secondary radicals generally inhibit removal efficiency [2]. Notably, the nonradical process seems to have better attributes for selective oxidation, and no or little inhibition can be found in certain systems [6,15,80]. To evaluate the influence of the water matrix, inorganic anions Cl^−^, NO_3_^−^, HCO_3_^−^, HPO_4_^2−^, and humic acid (sodium salt) were used. Tap water and actual surface water samples were applied to simulate practical conditions. Tests were carried out with background substances of 1 mM and 10 mM concentrations.

**Figure 6:**
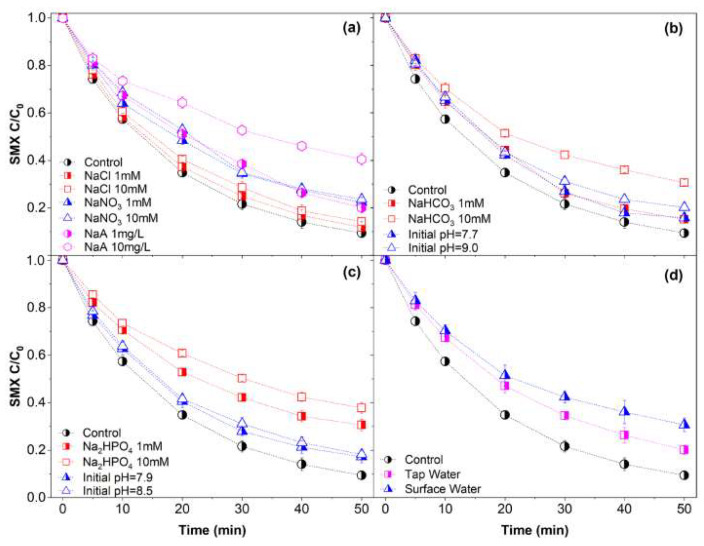
Effects of (**a**) Cl^−^, NO_3_^−^ and A^−^, (**b**) HCO_3_^−^, (**c**) HPO_4_^2^^−^ (**d**) tap water, surface water on SMX degradation by PDS/CuO@CNT. Conditions: SMX = 39.5 µM, PDS = 1.0 mM, CuO@CNT = 0.1 g/L.

As expounded in Figure 5a, Cl^−^ slightly inhibited the degradation efficiency. Researchers have claimed that some specific nonradical processes are not affected by Cl^−^ [6,81]. Cl• and HOCl were generated in the PMS system [17,82]. Therefore, in the surveyed system, reactions of Cl^−^ and weakly activated PDS on CuO may occur at a slow rate, and the generated secondary active species attacked SMX at a relatively low rate. NO_3_^−^ showed a slightly stronger inhibitory effect than Cl^−^, which was caused by competitive adsorption. No evidence can be found that secondary radicals might be formed. HCO_3_^−^ and HPO_4_^2−^ change the initial pH of the survey system, and thus, tests at the same initial pH were carried out to exclude the pH impact (shown in Figure 6b,c). No obvious suppression can be found with 1 mM NaHCO_3_. When the concentration of NaHCO_3_ was increased to 10 mM, the degradation efficiency of SMX decreased by 10.5%. In regard to Na_2_HPO_4_, the degradation efficiency decreased by 13.4% and 19.6% at 1 mM and 10 mM, respectively. $O_{2}^{\text{•}-}$ was consumed by HCO_3_^−^ [83] as shown in Equations (11)–(13), and therefore, the generation of ^1^O_2_ was inhibited. HPO_4_^2−^ may just occupy the active site as NO_3_^−^.

(11) $$O_{2}^{\text{•}-}{+\mathrm{HCO}}_{3}^{-}\;\to {\;\mathrm{CO}}_{3}^{\text{•}-}{+\mathrm{HO}}_{2}^{-}$$

(12) $$O_{2}^{\text{•}-}{+\mathrm{CO}}_{3}^{\text{•}-}\;\to {\;\mathrm{CO}}_{3}^{2-}{+O}_{2}$$

(13) $$O_{2}^{\text{•}-}{+\mathrm{CO}}_{3}^{2-}\;\to {\;\mathrm{CO}}_{3}^{\text{•}-}{+O}_{2}^{2-}$$

Humic sodium (NaA) demonstrated relatively severe inhibition (Figure 6a) since the macromolecules occupied the active sites and impeded the activation process [52]. As shown in Figure 6d, the degradation efficiency decreased by 10.7% and 21.2% in tap water and actual surface water, respectively (parameters are shown in Table S5). The results are mainly due to the reactions occurring on the surface of CuO@CNT by the water constituents. In general, all the inhibitions are at an acceptable level, indicating a positive prospect for practical applications. Still, competitive adsorption needs to be focused on.

### 2.5. Proposed Pathways

**Figure 7:**
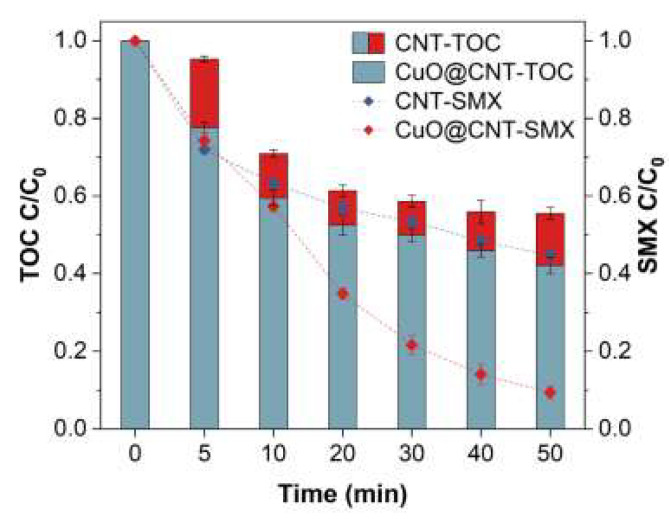
TOC removal of SMX degradation by PDS/CuO@CNT. Conditions: SMX = 39.5 µM, PDS = 1.0 mM, CuO@CNT = 0.1 g/L, pH = 5.6 ± 0.1.

The total organic carbon (TOC) removal in the surveyed system was evaluated. As illustrated in Figure 7, 58.0% of the TOC removal was achieved in the CuO@CNT/PDS/SMX system. Compared with the CNT/PDS/SMX system, the mineralization was increased by 13.4%. ^1^O_2_ was selective and the oxidation ability was relatively weaker than radicals. Adsorption cannot be ignored, as the intermediate products may be apt to be adsorbed by the catalyst. To further evaluate the degradation pathway, LC-MS and theoretical calculations were conducted. According to the Fukui functions, *f ^+^* represents electrophilic attack sites and *f ^−^* represents nucleophilic attack sites. The absolute values (shown in Table S6) can be used to predict the probability of reactions, and the visual models (Figure S15) show the contour surfaces of the values [84]. According to the calculation results, N and S are reactive sites.

**Figure 8:**
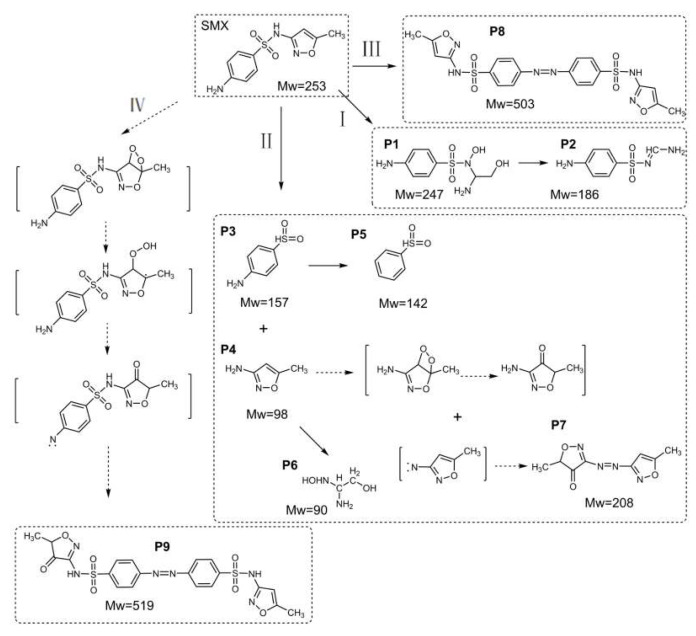
Pathway proposed for SMX degradation by PDS/CuO@CNT.

The intermediate products are detected by LC-MS (shown in Figure S16 and Table S7) and the proposed pathway is described in Figure 8. Pathway I is the ring-opening process of the isoxazole heterocycle, which seems to be a particular transition of the nonradical process in the PDS oxidation system [44]. Pathway II indicates the cleavage of the N-S bond, which is a typical SMX transformation [85,86,87]. Typical intermediate products P3 and P4 [88] are further converted to P5 and P6. Nitrogen-centered radicals are obtained during the direct oxidation of PDS, and then combined by contributing an electron [85]; thus, P8 is generated. As a dienophile, ^1^O_2_ is likely to react with olefins, dienes, and polycyclic aromatic compounds [10]. Electron-rich atoms in the isoxazole heterocycle are substrates for ^1^O_2_, and a carbonyl group finally forms as P9. P7 is composed of two nitrogen-centered isoxazole heterocycles, one of which is the adduct with ^1^O_2_. Toxic nitro-derivatives are not detected, although they do exist in some nonradical processes [45,89]. The proposed pathways indicate the unique oxidation products of SMX, particularly pathways III and Ⅳ.

## 3. Materials and Methods

### 3.1. Chemicals

CNTs were purchased from XFNANO Material Tech Co Ltd, Nanjing, China. Milli-Q ultrapure water (18.2 MΩ/cm) was employed throughout.

Methanol (MeOH), tert-butanol (TBA), furfuryl alcohol (FFA), p-benzoquinone (BQ), sodium azide (NaN_3_), peroxydisulfate (PDS), hydrogen peroxide (H_2_O_2_) and oxone (PMS, KHSO_5_•0.5KHSO_4_•0.5K_2_SO_4_), were purchased from Sigma-Aldrich, Shanghai, China. Sulfamethoxazole (SMX), ofloxacin (OFX), 2, 4-chlorophenol (2,4-DCP), p-chlorobenzene acid (p-CBA) and nitrobenzene (NB) were obtained from Aladdin Bio-Chem Technology Co., LTD., Shanghai, China. 5,5-Dimethyl-1-pyrroline N-oxide (DMPO) was purchased from Dojindo Molecular Technologies, INC., Japan. 2,2,6,6-Tetramethyl-4-piperidinyl (TMP) was purchased from Acros Organics, Belgium. Other chemicals were supplied by Sinopharm Chemical Reagent Co., Ltd., Shanghai, China.

### 3.2. Catalyst Preparation and Characterization

CNT-supported CuO was synthesized according to the previous literature [42]. Carboxylated CNTs (acidized by nitric acid) were ultrasonically dispersed for 30 min. Excessive ammonium hydroxide was added dropwise into a copper acetate solution; then, the blue complex solution and CNT dispersion liquid were diffused, and the mixture was continuously magnetically stirred for some time. After filtration, the suspension was washed to a neutral pH, vacuum freeze-dried, and then, heated up to 400 °C and kept for 2 h in a tube furnace in an argon atmosphere. The obtained catalyst was named CuO@CNT. Samples with different copper contents were prepared. CuO was obtained by the calcination of copper acetate at 400 °C.

Details of the characterizations are shown in the Supplementary Materials (Text S1).

### 3.3. Experimental Setup and Procedure

All reactions were conducted in a crystallizing dish by magnetic stirring with a total reaction volume of 200 mL, and the temperature was kept at 25 °C. Bulk solutions contained the target organic pollutants, PDS (or PMS, H_2_O_2_), catalyst, and chemical quencher if needed. The pH was adjusted using 0.1 M sulfuric acid and 0.1 M sodium hydroxide. At a given time, a sample was withdrawn and filtered with a 0.22 μM filter (polytetrafluoroethylene syringe filters), and then, quenched immediately with excess sodium thiosulfate. Three parallel experiments were carried out for precision.

### 3.4. Analytic Methods

All the pH values in this paper indicated a steady pH value after the addition of PDS.

To detect reactive oxygen species, EPR spectroscopy was conducted with 2,2,6,6-tetramethyl-4-piperidone (TEMP) and 5,5-dimethyl-1-pyrroline-N-oxide (DMPO) as spin-trapping reagents in water or DMSO. Cu(III) was detected via the UV–vis spectrum.

Cyclic voltammetry (CV) measurements were conducted. A standard three-electrode cell system (a glassy carbon electrode as the working and counter electrode, a Ag/AgCl electrode as the reference electrode, and a 0.5 M sodium sulfate as the electrolyte) was connected with a CHI 700E electrochemical workstation in the potential region from −0.5 V to 1.0 V at a scan rate of 10 mV/s.

The concentrations of contaminants, PDS, TOC, and Cu^2+^, were detected. The methods are provided in the Supplementary Materials (Text S2). The intermediates were further analyzed using high-performance liquid chromatography–mass spectrometry (LC-MS, Waters 2695, Thermo Fisher LCQTM Deca XP plus) equipped with an electrospray ionization (ESI) source. Details are provided in the Supplementary Materials (Text S3).

## 4. Conclusions

To sum up, we proposed a simple strategy to remove SMX through a selective oxidation process. It is a pioneering approach to employing CuO@CNT as a catalyst for PDS. The simultaneous production of ^1^O_2_ and activated PDS as the ultimate oxidative species was developed. PDS not only acted as oxidation attributed to the heterogeneous surface activation, but also contributed oxygen in the reaction, generating ^1^O_2_ with the in situ Cu(III). Cu(III) was detected, though, and involved as a metastable intermediate rather than as a direct oxidant for SMX. DO also contributed to the generation of a relatively small portion of ^1^O_2_. Moreover, the substrate-dependent mechanism indicates that it may be practically used for the removal of electron-rich pollutants, such as antibiotics and chlorophenol. The system was mildly affected by background substances, i.e., Cl^−^ and HCO_3_^−^. Nine intermediate products were derived from the breakage of the N-S bond, the ring-opening reaction of the heterocycle, the combination of N-centered radicals, and electrophilic addition by ^1^O_2_. Unlike the radical process, toxic nitro-products were not detected. The characterizations and cycle tests proved the satisfactory stability of CuO@CNT. Overall, this research broadened the application of the nonradical process, especially in the process of Cu-based material catalytic PDS oxidation.

## Data Availability

Not applicable.

## Supplementary Materials

The following supporting information can be downloaded at: https://www.mdpi.com/article/10.3390/molecules27207064/s1, Figure S1: XRD patterns of CuO@CNT with different copper content; Figure S2: BET measurement for CuO; Figure S3: FTIR spectra of CNT, CuO@CNT and CuO; Figure S4: SMX degradation in the PDS system activated by the CuO@CNT with different cooper loading; Figure S5: SMX degradation and the ionic leaching in the system of PMS, H2O2, and PDS; Figure S6: SMX degradation in Cu2+/PDS system; Figure S7: Adsorption and degradation of NB, p-CBA, FFA, SMX, OFX, 2,4-DCP by CuO@CNT; Figure S8: Degradation kinetics of the different targets; Figure S9: EPR spectra of •OH and SO_4_^•−^; Figure S10: Degradation efficiency of SMX and PDS; Figure S11: Reusability of CuO@CNT for SMX degradation; Figure S12: Zeta potential of CuO@CNT at different pH conditions; Figure S13: (a) pH effect on SMX degradation by CuO@CNT/PDS, (b) SMX distribution at different pH; Figure S14: Degradation kinetics of SMX influenced by dosages of SMX, catalyst, and PDS; Figure S15: Visualized isosurface of Fukui function on SMX; Figure S16: LC-MS chromatograms of SMX and the transition products; Figure S17: Numbers of the atoms in SMX; Table S1: Reaction rate constants of the chemical quenchers and ROS [4,90]; Tables S2–S4: Pseudo-first order parameters of SMX degradation influenced by dosages of SMX, catalyst, and PDS; Table S5: Parameters of tap water and surface water; Table S6: Fukui function values of the SMX atoms; Table S7: Intermediate products proposed; Text S1: Characterization methods; Text S2: Concentration measurements; Text S3: LC-MS analytic methods for intermediate products.
