# Supplementary material for: Enhanced Degradation of Antibiotic by Peroxydisulfate Catalysis with CuO@CNT: Simultaneous 1O2 Oxidation and Electron-Transfer Regime
Source: Molecules. 2022 Oct 19;27(20):7064. doi: 10.3390/molecules27207064 (PMC9609598; doi:10.3390/molecules27207064)
Supplement: Supplementary file 1 [file molecules-27-07064-s001.zip › molecules-1929133-supplementary.pdf]

Supplementary Material:

Enhanced degradation of antibiotic by peroxydisulfate catalysis with CuO@CNT: simultaneous $^1O_2$ oxidation and electron-transfer regime

Jia Liu, Chao Ding, Sicheng Gong, Kun Fu, Jun Shi*, Huiping Deng*

Key Laboratory of Yangtze River Water Environment, Ministry of Education, Shanghai Institute of Pollution Control and Ecological Security, Shanghai 200092, P.R. China.

State Key Laboratory of Pollution Control and Resources Reuse, College of Environmental Science and Engineering, Tongji University, Shanghai 200092, P. R. China.

E-mail addresses:

shijun215@tongji.edu.cn (J. Shi), oneenvironscitec@outlook.com (H.P. Deng)

*Author to whom correspondence should be addressed

Huiping Deng, E-mail: oneenvironscitec@outlook.com; Phone: +86-021-65982688.

Jun Shi, E-mail: shijun215@tongji.edu.cn; Phone: +86-021-65982688.

Totally 31 pages including 3 texts, 7 tables, and 17 figures.

Text S1. Characterization methods.

Text S2. Concentration measurements.

Text S3. LC-MS analytic methods for intermediate products.

Table S1. Reaction rate constants of the chemical quenchers and ROS.

Table S2-S4. Pseudo-first order parameters of SMX degradation influenced by dosages of SMX, catalyst, and PDS.

Table S5. Parameters of tap water and surface water.

Table S6. Fukui function values of the SMX atoms.

Table S7. Intermediate products proposed.

Figure S1. XRD patterns of CuO@CNT with different copper content.

Figure S2. BET measurement for CuO.

Figure S3. FTIR spectra of CNT, CuO@CNT and CuO.

Figure S4. SMX degradation in the PDS system activated by the CuO@CNT with different cooper loading.

Figure S5. SMX degradation and the ionic leaching in the system of PMS, H_2O_2, and PDS.

Figure S6. SMX degradation in Cu^{2+}/PDS system.

Figure S7. Adsorption and degradation of NB, *p*-CBA, FFA, SMX, OFX, 2,4-DCP by CuO@CNT.

Figure S8. Degradation kinetics of the different targets. Conditions: [Target] = 39.5μM, [PDS] = 1.0 mM, [CuO@CNT] = 0.1 g/L, pH = 5.6 ± 0.1.

Figure S9. EPR spectra of •OH and $SO_4^{\bullet -}$.

Figure S10. Degradation efficiency of SMX and PDS.

Figure S11. Reusability of CuO@CNT for SMX degradation.

Figure S12. Zeta potential of CuO@CNT at different pH conditions.

Figure S13. (a) pH effect on SMX degradation by CuO@CNT/PDS, (b) SMX distribution at different pH. Conditions: [SMX] = 39.5 μM, [PDS] = 1.0 mM, [CuO@CNT] = 0.1 g/L.

Figure S14. Degradation kinetics of SMX influenced by dosages of SMX, catalyst, and PDS.

Figure S15. Visualized isosurface of Fukui function on SMX.

Figure S16. LC-MS chromatograms of SMX and the transition products.

Figure S17. Numbers of the atoms in SMX.

Text S1. Characterization methods.

XRD (Brüker D8 Advance diffractometer) using Cu Ka radiation was employed to detect the crystalline structures of the catalysts. The morphology was observed using a scanning electron microscope (Hitachi S4800) and transmission electron microscope (TFEI TecnaiG2 F20 S-Twin). N_2 adsorption-desorption measurements were conducted using an accelerated surface area and porosity analyzer (ASAP 2460, Micromeritics). The XPS data were recorded on a Kratos AXIS Supra instrument (Kratos Analytical, Manchester, UK) using monochromatic Al Ka radiation (1486.7 eV, 150W). The zeta potential was analyzed using a Nano Particle Potentiometer (Zetasizer Nano Zs90, Malvern). FTIR spectra of the composites were recorded using a Thermo Fisher DTGS spectrometer.

Text S2. Concentrations measurements.

The concentrations of NB, *p*-CBA, FFA, OFX, SMX, and 2,4-DCP were measured by a reverse-phase high-performance liquid chromatography system (Agilent 1200) equipped with an Agilent ZORBAX Eclipse Plus C18 column (4.6×250 mm, 5 μm) and a diode array detector (DAD) at wavelengths of 270,234, 220, 288, 260, 285nm, respectively. The mobile phase consists of methanol and water containing 0.1% formic acid. The flow rate was 1 mL/min and the injected volume was 50 μL.

The concentration of PDS was measured by the spectrophotometric method suggested by Liang. Briefly, a high concentration solution of iodide (KI 0.5 M) with bicarbonate buffer ($NaHCO_3$ 0.05 M) was prepared first, and then 0.1 mL filtered

sample was mixed with the above iodide solution (4.9 mL). The chromogenic sample was detected at λ = 352 nm (UV-Vis spectrophotometer, Thermo) in one minute.

The total organic carbon (TOC) was measured by a multi N/C 3100 analyzer (Analytikjena). pH values were measured by a METTLER TOLEDO pH meter. The mass of Cu^{2+} was measured by inductively coupled plasma optical emission spectrometry (ICP-OES, Optima 8000, Perkin Elmer).

Text S3. LC-MS analytic methods for intermediate products.

The intermediates were further analyzed using a high-performance liquid chromatography-mass spectrum (LC-MS, Waters 2695-ThermoFisher LCQTM Deca XP plus) equipped with an Electrospray ionization (ESI) source. The mobile phases consisted of water with 0.1% formic acid (A) and acetonitrile with 0.1% formic acid (B), and the chromatographic separations were performed with a 75mm× 2mm Luna 3μm C18 column. MS analyses were conducted with both positive and negative modes (ESI+/ESI-) over a mass scan range of 50-400 m/z. For the chromatographic separation, a gradient elution method with two mobile phases was employed as follows: 90% A for 0-0.5 min; 90% A decreased to 10% A lineally for 0.5-5.5 min and kept for 5.5-8.0 min; 10% A increased to 90% A lineally for 8.0-9.5 min and kept for 9.5-10.0 min. The flow rate was kept at 0.2 mL/min.

Table S1. Reaction rate constants of the chemical quenchers and ROS[1, 2].

Quenching	$k_{\bullet OH}$ ($M^{-1}s^{-1}$)	$k_{SO_4^{\bullet -}}$ ($M^{-1}s^{-1}$)	k_{1O_2}($M^{-1}s^{-1}$)	$k_{\bullet O_2^-}$($M^{-1}s^{-1}$)
TBA	6×10^8	7.6×10^5	1.8×10^3	Unknown
MeOH	9.7×10^8	2.5×10^7	3×10^3	Unknown
NB	3.9×10^9	$<10^6$	Unknown	Unknown
BQ	Unknown	Unknown	Unknown	2.9×10^9
FFA	1.5×10^{10}	Unknown	1.2×10^8	Unknown
NaN_3	1.2×10^{10}	2.5×10^9	1.0×10^9	Unknown
p-CBA	5.0×10^9	3.8×10^8	Unknown	Unknown

Table S2. SMX degradation (%) suppressed in quenching tests.

Quencher	Air	N_2	difference
Control	90.60	75.40	15.20
MeOH (100mM)	–	–	–
TBA (100mM)	8.03	4.83	3.20
NaN_3 (10mM)	30.90	15.50	15.40
BQ (10mM)	42.40	23.50	18.90

Table S3-5. Pseudo-first order parameters of SMX degradation influenced by dosages of SMX, catalyst, and PDS.

Table S3. Pseudo-first order parameters of SMX degradation influenced by dosage of SMX. Conditions: [PDS] = 1 mM, [CuO@CNT] = 0.1 g/L, pH = 5.6 ± 0.1.

C_{SMX}(μM)	K(s^{-1})	R^2	Standard error of K
19.8	0.0808	0.998	1.54×10^{-3}
39.5	0.0472	0.997	1.12×10^{-3}
79	0.0137	0.976	8.83×10^{-4}
158	0.00713	0.912	8.96×10^{-4}

Table S4. Pseudo-first order parameters of SMX degradation influenced by dosage of CuO@CNT. Conditions: [PDS] = 1 mM, [SMX] = 39.5 μM, pH = 5.6 ± 0.1.

$C_{catalyst}$(gL^{-1})	K(s^{-1})	R^2	Standard error of K
0.5	0.0128	0.994	3.81×10^{-4}
1	0.0472	0.997	1.12×10^{-3}
2	0.122	0.972	1.88×10^{-4}
4	0.219	0.999	4.65×10^{-3}

Table S5. Pseudo-first order parameters of SMX degradation influenced by dosage of PDS. Conditions: [CuO@CNT] = 0.1 g/L, [SMX] = 39.5 μM, pH = 5.6 ± 0.1.

C_{PDS}(mM)	K(s^{-1})	R^2	Standard error of K
0.5	0.0450	0.994	1.40×10^{-3}
1	0.0472	0.997	1.12×10^{-3}
2	0.0493	0.998	9.54×10^{-4}
4	0.0569	0.995	1.65×10^{-3}

Table S6. Parameters of tap water and surface water.

Constituent	Tap water	Surface water
pH	7.78	7.58
DOC (mg L^{-1})	1.08	3.81
UV_{254} (cm^{-1})	0.043	0.14
CO_3^{2-} (mg L^{-1})	0.83	1.89
HCO_3^- (mg L^{-1})	104	150
Cl^- (mg L^{-1})	6.6	13.1
SO_4^{2-} (mg L^{-1})	104.1	150.1
PO_4^{3-} (mg L^{-1})	1.63	4.35
NO_3^- (mg L^{-1})	2.03	1.54
Ammonia (mg-N L^{-1})	-	0.35

Table S7. Fukui function values of the SMX atoms.

Atom	*f*-	*f*+
1 (C)	0.0215	0.0249
2 (C)	0.0134	0.0095
3 (C)	0.0333	0.0348
4 (O)	0.0438	0.0477
5 (H)	0.0135	0.0122
**6 (N)**	**0.0763**	0.0643
**7 (N)**	**0.0646**	0.0532
8 (H)	0.0223	0.0282
9 (C)	0.0129	0.0131
10 (H)	0.0185	0.0192
11 (H)	0.0151	0.0160
12 (H)	0.0131	0.0129
13 (S)	0.0167	**0.0931**
14 (O)	0.0519	0.0766
15 (O)	0.0453	0.0702
16 (C)	0.0529	0.0287
17 (C)	0.0295	0.0395
18 (C)	0.0269	0.0413
19 (C)	0.0522	0.0333
20 (H)	0.0213	0.0231
21 (C)	0.0541	0.0308
22 (H)	0.0190	0.0216
23 (C)	0.0413	0.0584
24 (H)	0.0284	0.0271
25 (H)	0.0279	0.0258
**26 (N)**	**0.1085**	0.0452
27 (H)	0.0381	0.0249
28 (H)	0.0376	0.0245

Table S8. Intermediate products proposed.

Degradation products	Retention time (min)	Proposed Formula	Observed M/Z	Structural formula
SMX	6.12	$C_{10}H_{11}N_3O_3S$	254.31	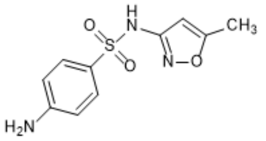
P1	1.35	$C_8H_{12}N_3O_4S$	249.14	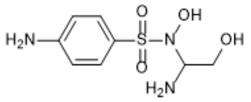
P2	5.65	$C_7H_8N_3O_2S$	186.38	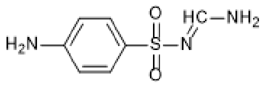
P3	1.19	$C_6H_7NO_2S$	159.23	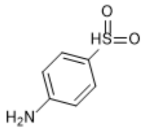
P4	3.05	$C_4H_6N_2O$	99.32	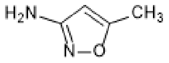
P5	1.05	$C_6H_6O_2S$	143.26	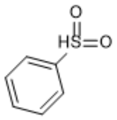
P6	1.23	$C_2H_8N_2O_2$	91.26	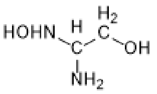
P7	1.00	$C_8H_7N_4O_3$	208.26	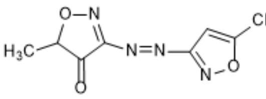
P8	1.25	$C_{20}H_{18}N_6O_6S_2$	505.33	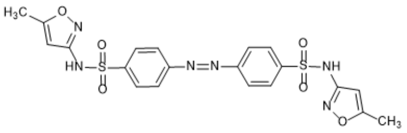
P9	5.30	$C_{20}H_{18}N_6O_7S_2$	520.76	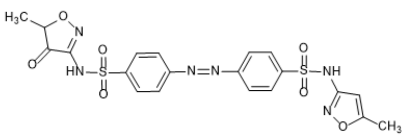

Figure S1. XRD patterns of CuO@CNT with different copper content (wt%).

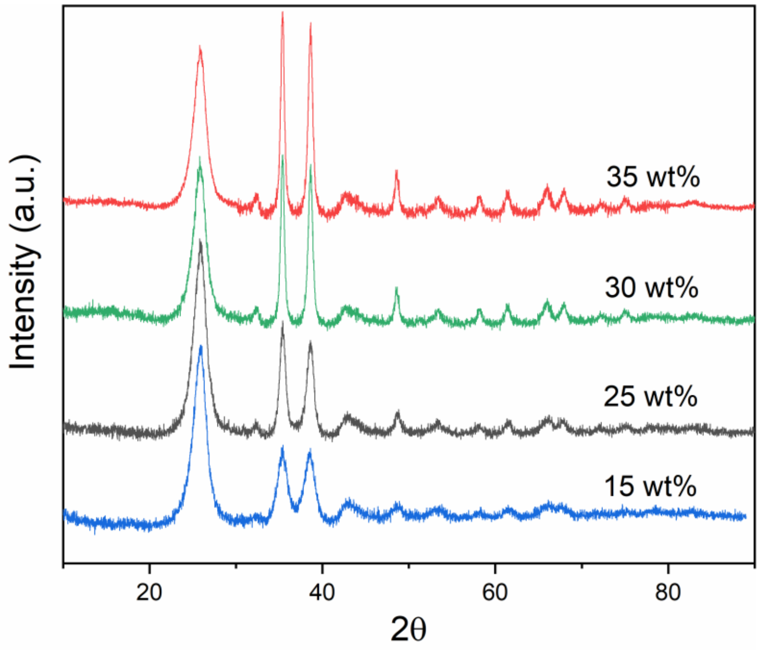

Figure S2. BET measurement for CuO.

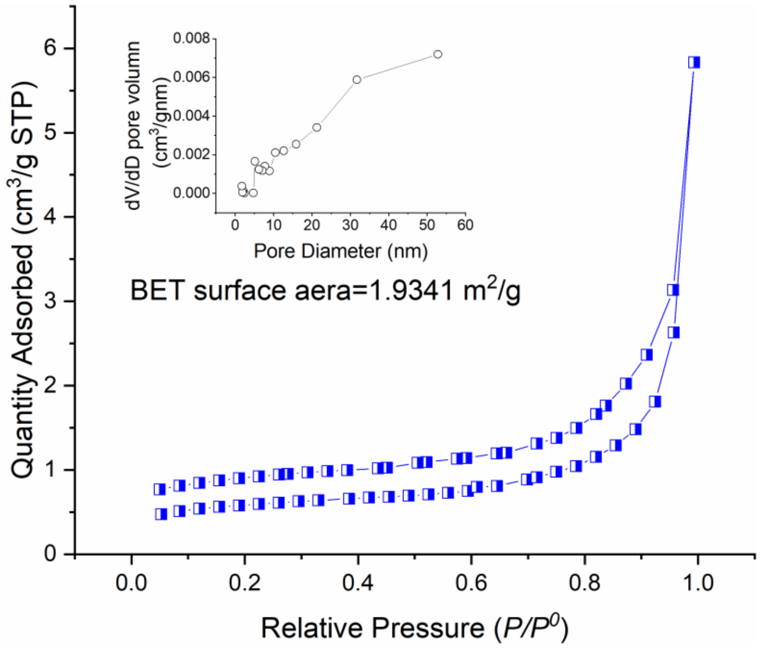

Figure S3. FTIR spectra of CNT, CuO@CNT and CuO.

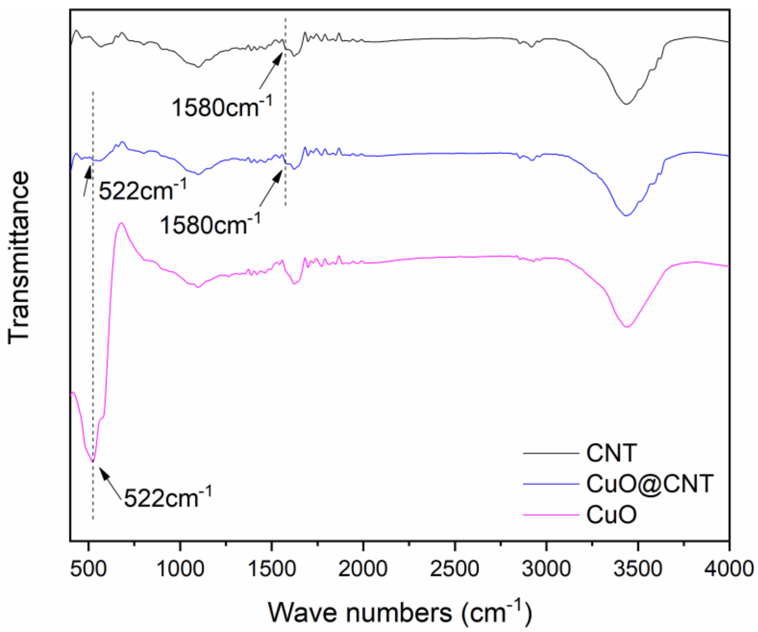

Figure S4. SMX degradation in the PDS system activated by the CuO@CNT with different cooper loading. Conditions: [SMX] = 39.5 μM, [CuO@CNT] = 0.1 g/L, pH = 5.6 ± 0.1, [Oxidant] = 1.0 mM.

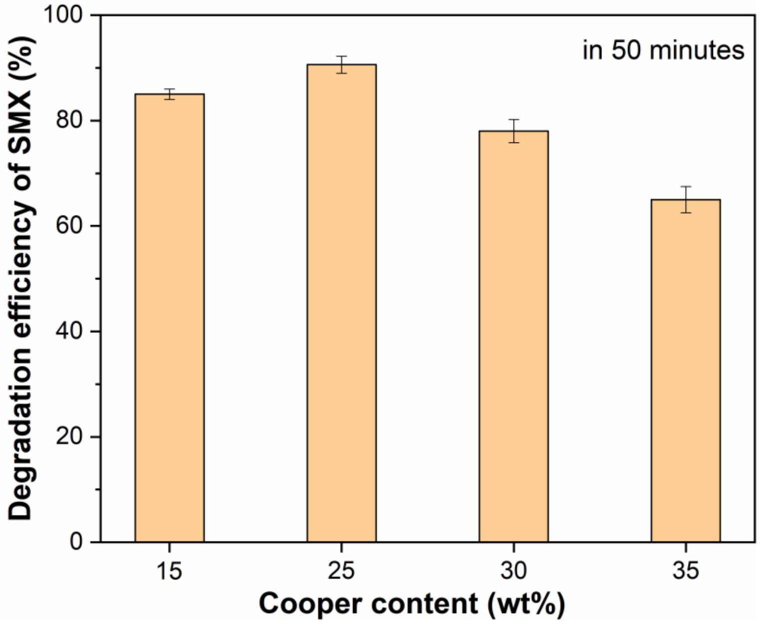

Figure S5. SMX degradation and the ionic leaching in the system of PMS, H_2O_2, and PDS. Conditions: [SMX] = 39.5 μM, [CuO@CNT] = 0.1 g/L, pH = 5.6 ± 0.5, [Oxidant] = 1.0 mM.

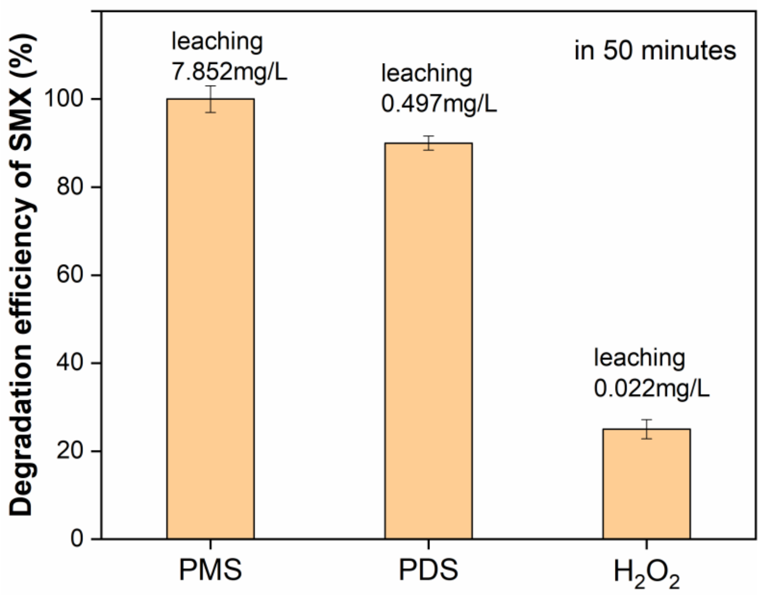

Figure S6. SMX degradation in Cu^{2+}/PDS system. Conditions: [SMX] = 39.5 μM, [PDS] = 1.0 mM, pH = 5.6 ± 0.1.

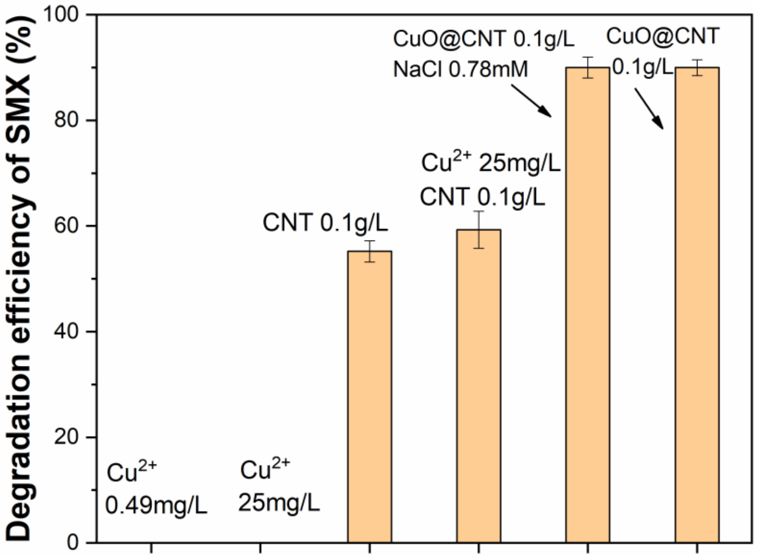

Figure S7. Adsorption and degradation efficiencies (%) of NB, *p*-CBA, FFA, SMX, OFX, 2,4-DCP by CuO@CNT. Conditions: [Contanminant] = 39.5 μM, [PDS] = 1.0 mM, pH = 5.6 ± 0.1.

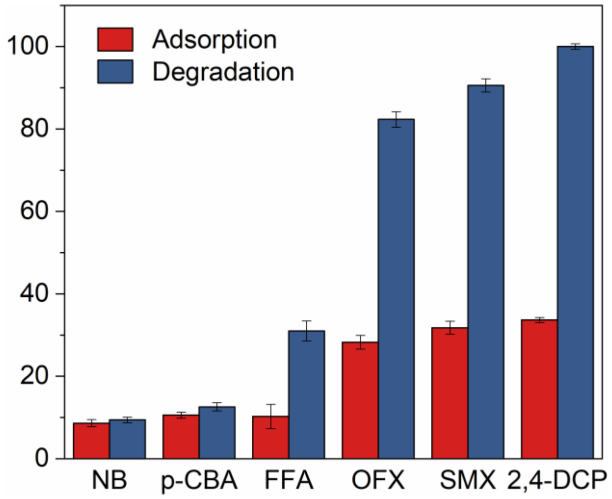

Figure S8. Degradation kinetics of the different targets. Conditions: [Target] = 39.5μM, [PDS] = 1.0 mM, [CuO@CNT] = 0.1 g/L, pH = 5.6 ± 0.1.

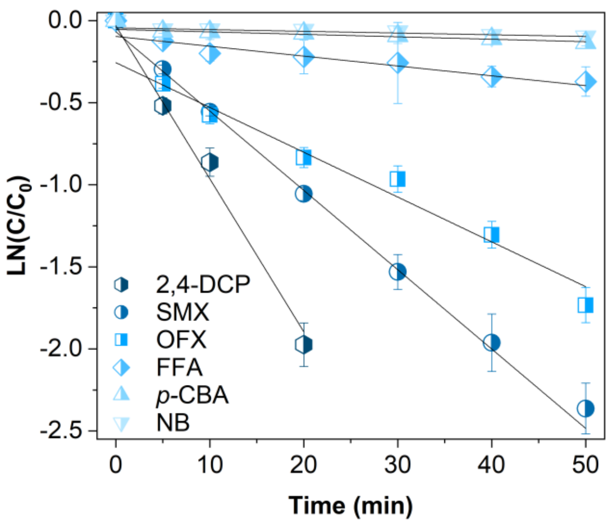

Figure S9. EPR spectra of •OH and $SO_4^{\bullet-}$. Conditions: [PDS] = 1.0 mM, [CuO@CNT] = 0.1 g/L, [MeOH] = 100mM, [TBA] = 100mM.

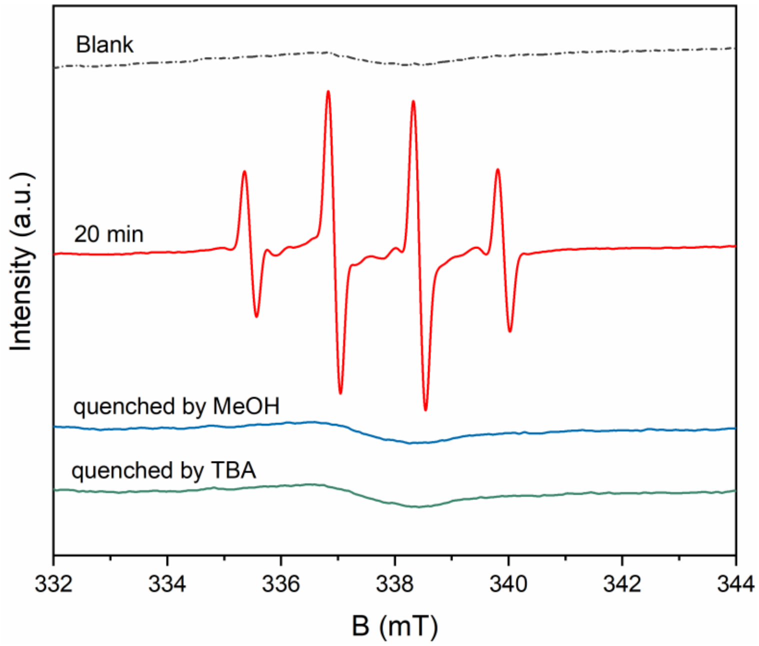

Figure S10. Degradation efficiency of SMX and PDS. Conditions: [SMX] = 39.5 μM, [PDS] = 1.0 mM, [CuO@CNT] = 0.1 g/L, $[[Cu(HIO_6)_2]^{5-}]$ = 0.40 mM, pH = 13 ± 0.1.

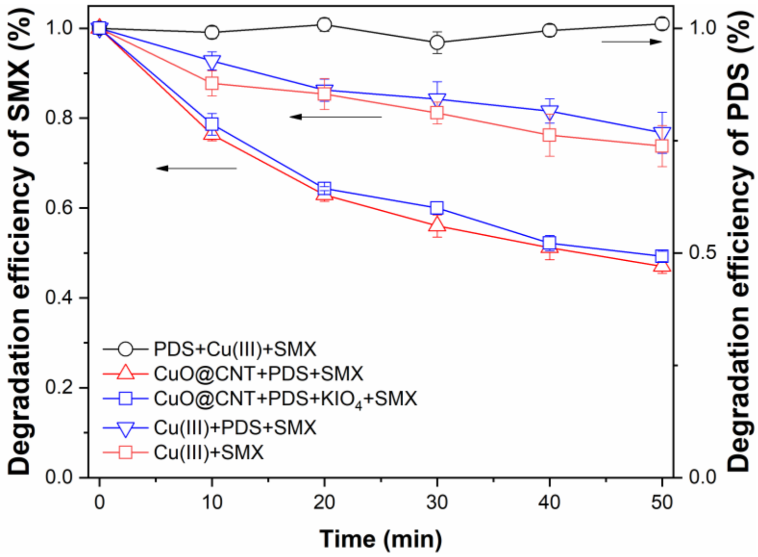

Figure S11. Reusability of CuO@CNT for SMX degradation. Conditions: [SMX] = 39.5 μM, [PDS] = 1.0 mM, [CuO@CNT] = 0.1 g/L, pH = 5.6 ± 0.1.

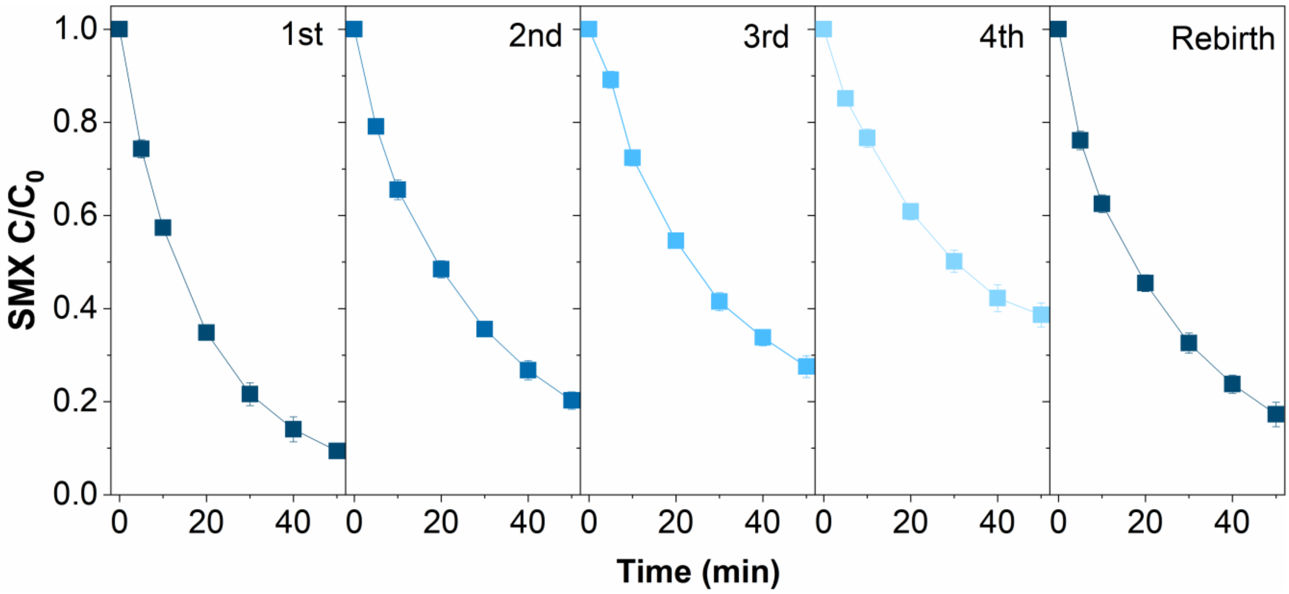

Figure S12. Zeta potential of CuO@CNT at different pH conditions.

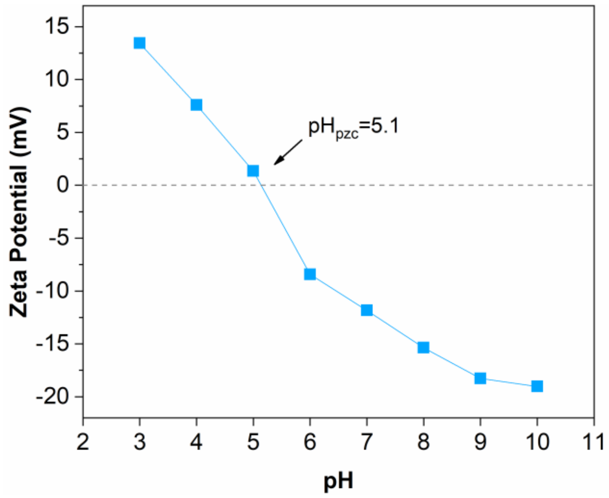

Figure S13. (a) pH effect on SMX degradation by CuO@CNT/PDS, (b) SMX distribution at different pH. Conditions: [SMX] = 39.5 μM, [PDS] = 1.0 mM, [CuO@CNT] = 0.1 g/L.

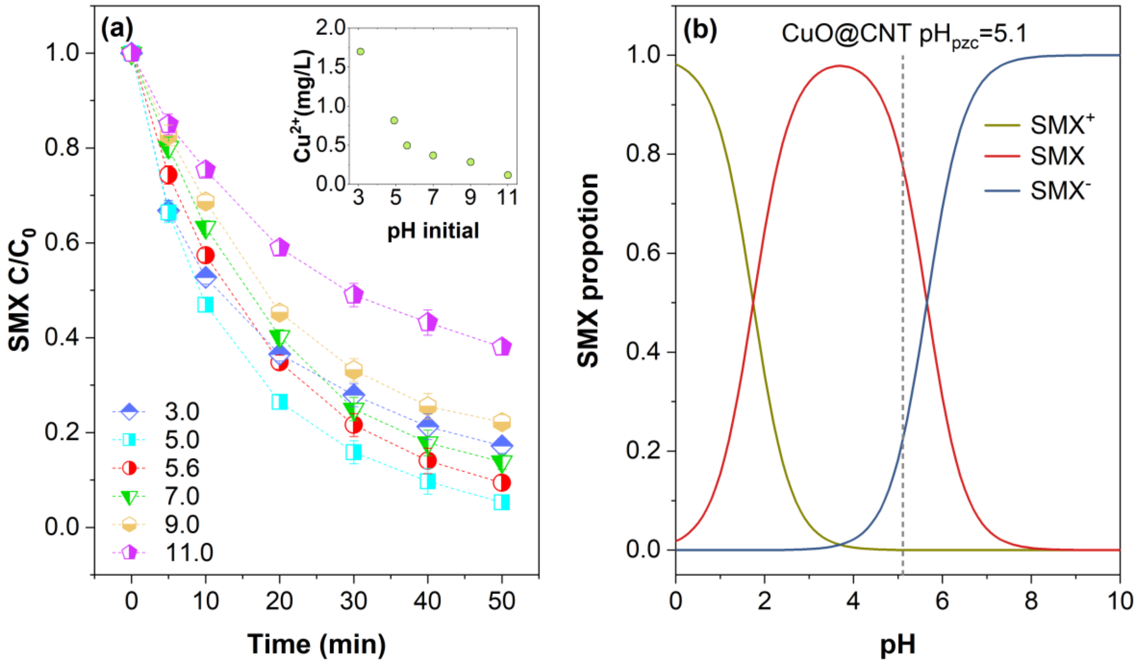

Figure S14. Degradation kinetics of SMX (a) influenced by dosage of SMX, [PDS] = 1.0 mM, [CuO@CNT] = 0.1 g/L, (b) influenced by dosage of catalyst, [SMX] = 39.5μM, [PDS] = 1.0 mM, and (c) influenced by dosage of PDS, [SMX] = 39.5 μM, [CuO@CNT] = 0.1 g/L.

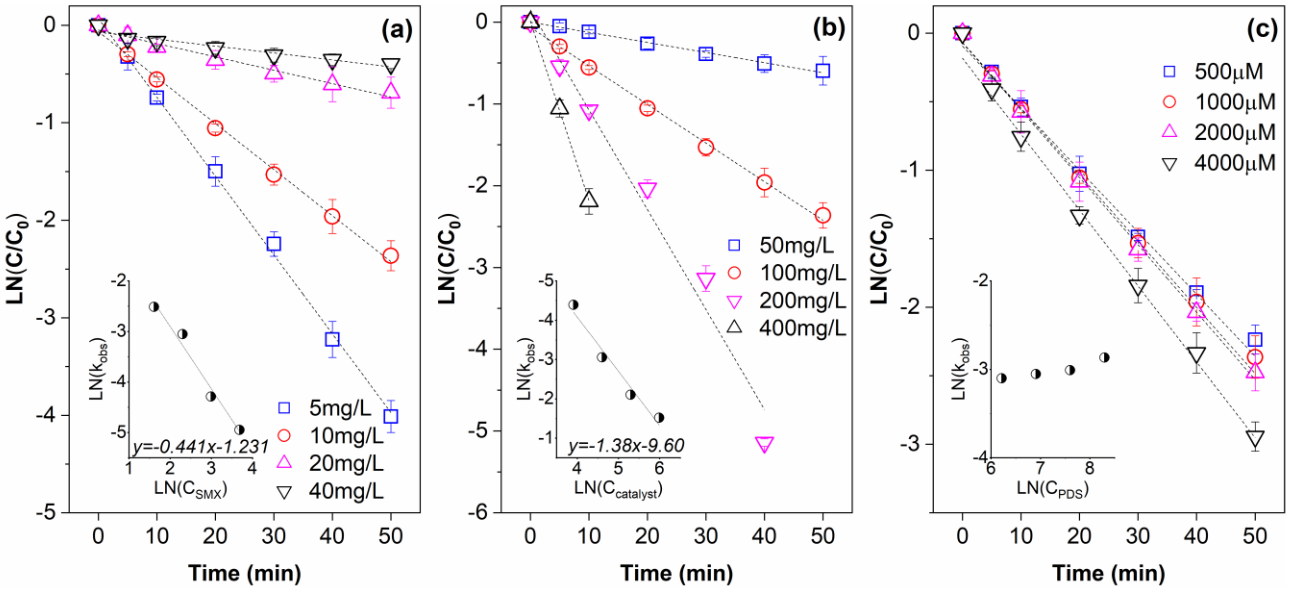

Figure S15. Visualized isosurface of Fukui function on SMX.

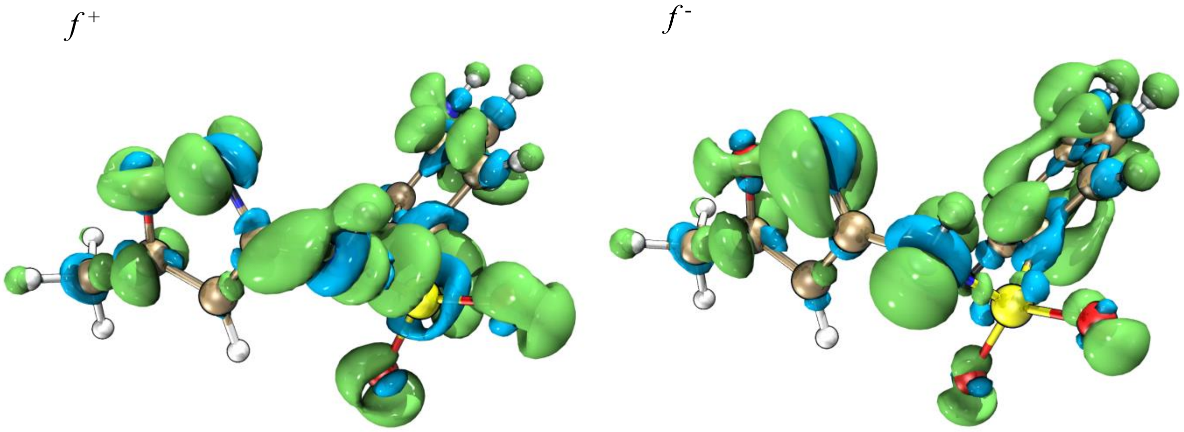

Figure S16. LC-MS chromatograms of SMX and the transition products.

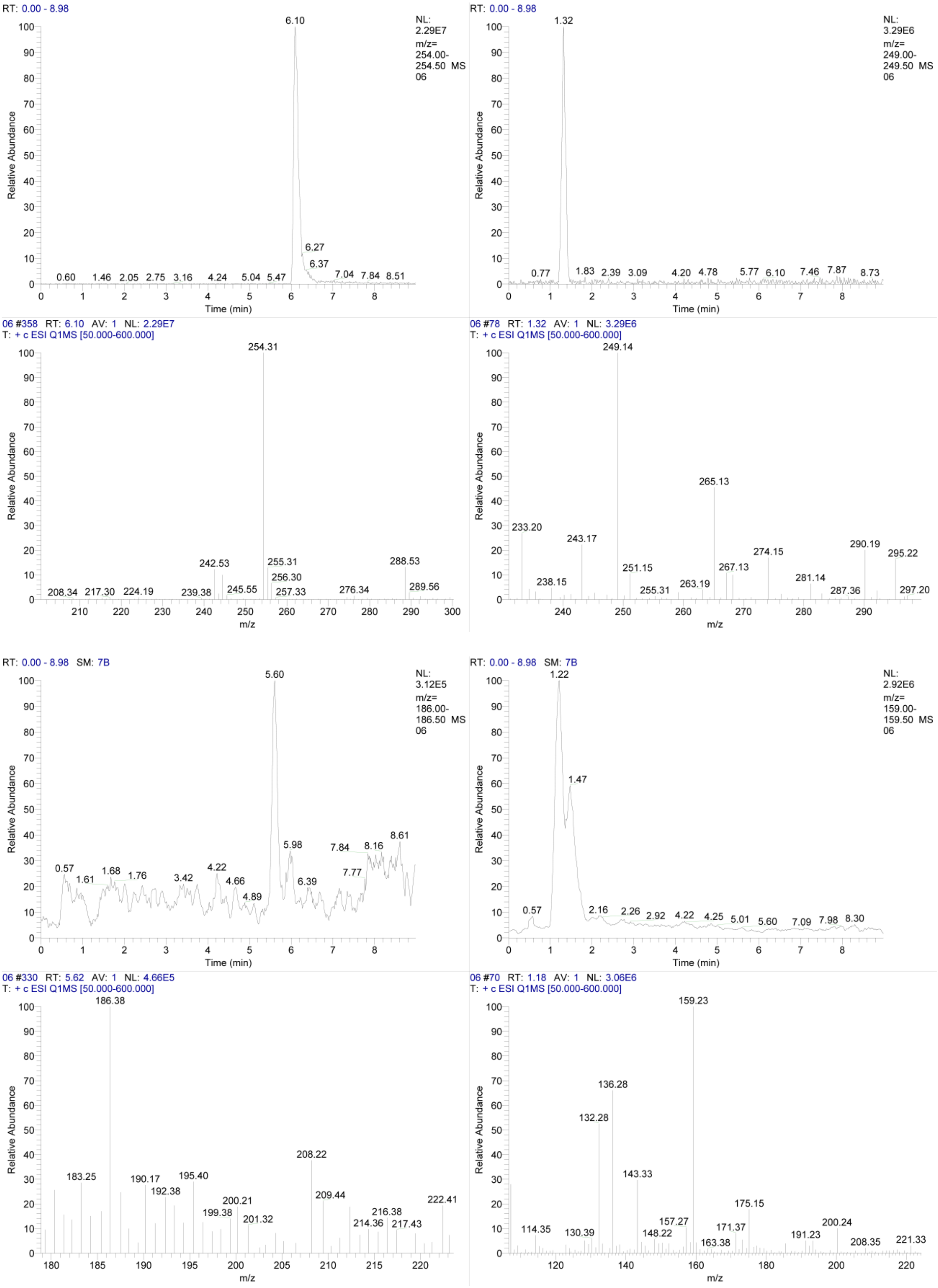

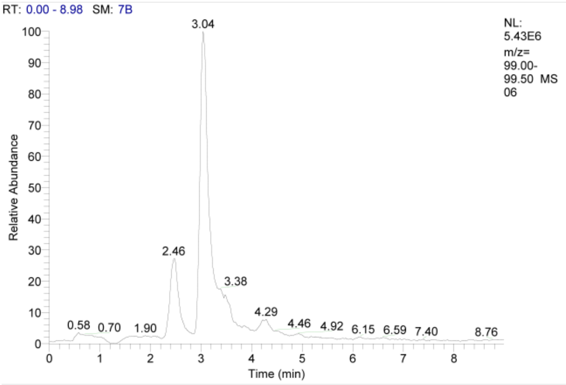
RT: 0.00 - 8.98 SM: 7B
NL: 5.43E6 m/z= 99.00-99.50 MS 06
Relative Abundance
Time (min)
3.04
2.46
3.38
4.29
0.58 0.70 1.90
4.46 4.92 6.15 6.59 7.40 8.76

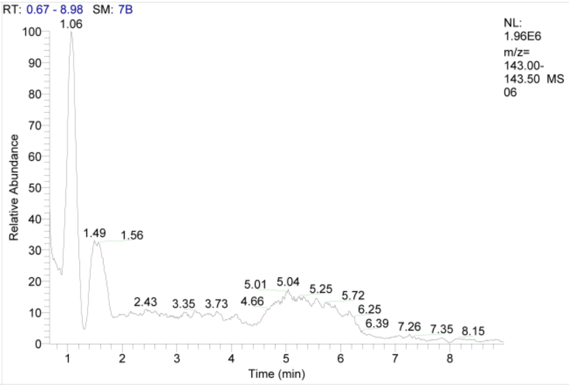
RT: 0.67 - 8.98 SM: 7B
NL: 1.96E6 m/z= 143.00-143.50 MS 06
Relative Abundance
Time (min)
1.06
1.49 1.56
2.43 3.35 3.73 4.66 5.01 5.04 5.25 5.72 6.25 6.39 7.26 7.35 8.15

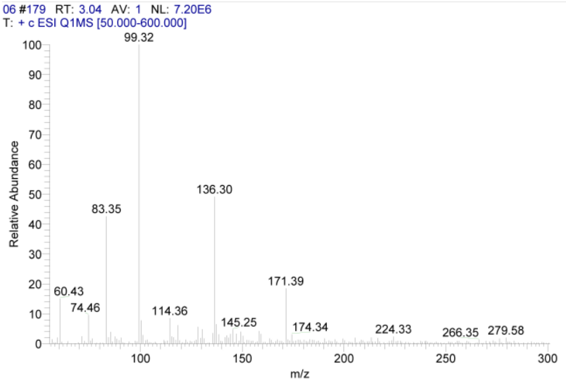
06 #179 RT: 3.04 AV: 1 NL: 7.20E6
T: + c ESI Q1MS [50.000-600.000]
Relative Abundance
m/z
99.32
136.30
83.35
171.39
60.43 74.46 114.36 145.25 174.34 224.33 266.35 279.58

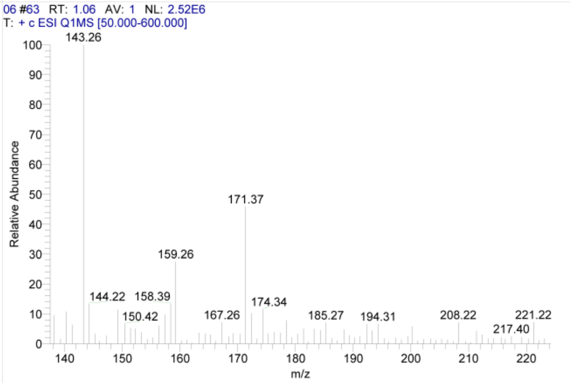
06 #63 RT: 1.06 AV: 1 NL: 2.52E6
T: + c ESI Q1MS [50.000-600.000]
Relative Abundance
m/z
143.26
171.37
159.26
144.22 158.39 150.42 167.26 174.34 185.27 194.31 208.22 217.40 221.22

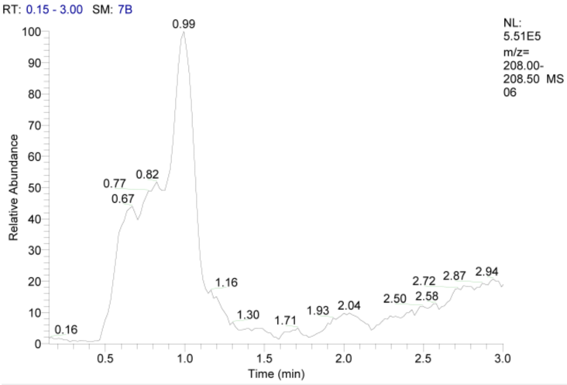
RT: 0.15 - 3.00 SM: 7B
NL: 5.51E5 m/z= 208.00-208.50 MS 06
Relative Abundance
Time (min)
0.99
0.77 0.82 0.67
1.16
0.16 1.30 1.71 1.93 2.04 2.50 2.58 2.72 2.87 2.94

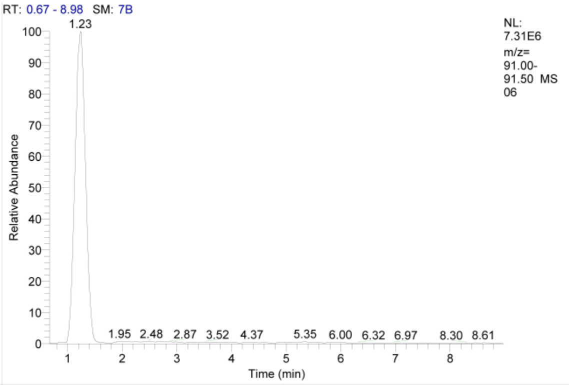
RT: 0.67 - 8.98 SM: 7B
NL: 7.31E6 m/z= 91.00-91.50 MS 06
Relative Abundance
Time (min)
1.23
1.95 2.48 2.87 3.52 4.37 5.35 6.00 6.32 6.97 8.30 8.61

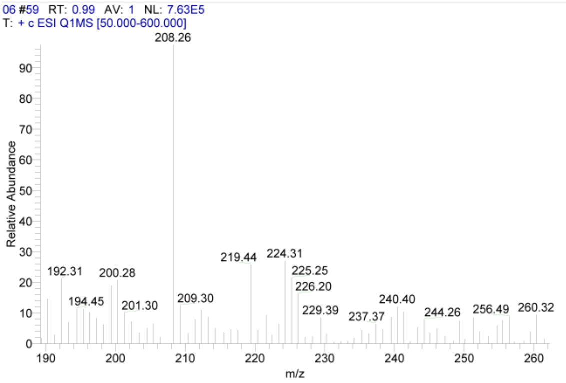
06 #59 RT: 0.99 AV: 1 NL: 7.63E5
T: + c ESI Q1MS [50.000-600.000]
Relative Abundance
m/z
208.26
219.44 224.31
192.31 200.28 225.25 226.20
194.45 201.30 209.30 229.39 237.37 240.40 244.26 256.49 260.32

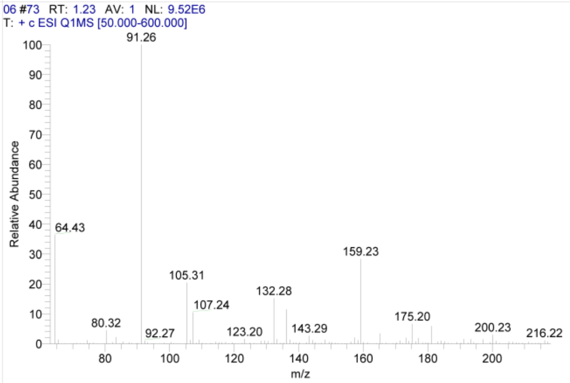
06 #73 RT: 1.23 AV: 1 NL: 9.52E6
T: + c ESI Q1MS [50.000-600.000]
Relative Abundance
m/z
91.26
64.43
159.23
105.31 132.28
107.24 80.32 92.27 123.20 143.29 175.20 200.23 216.22

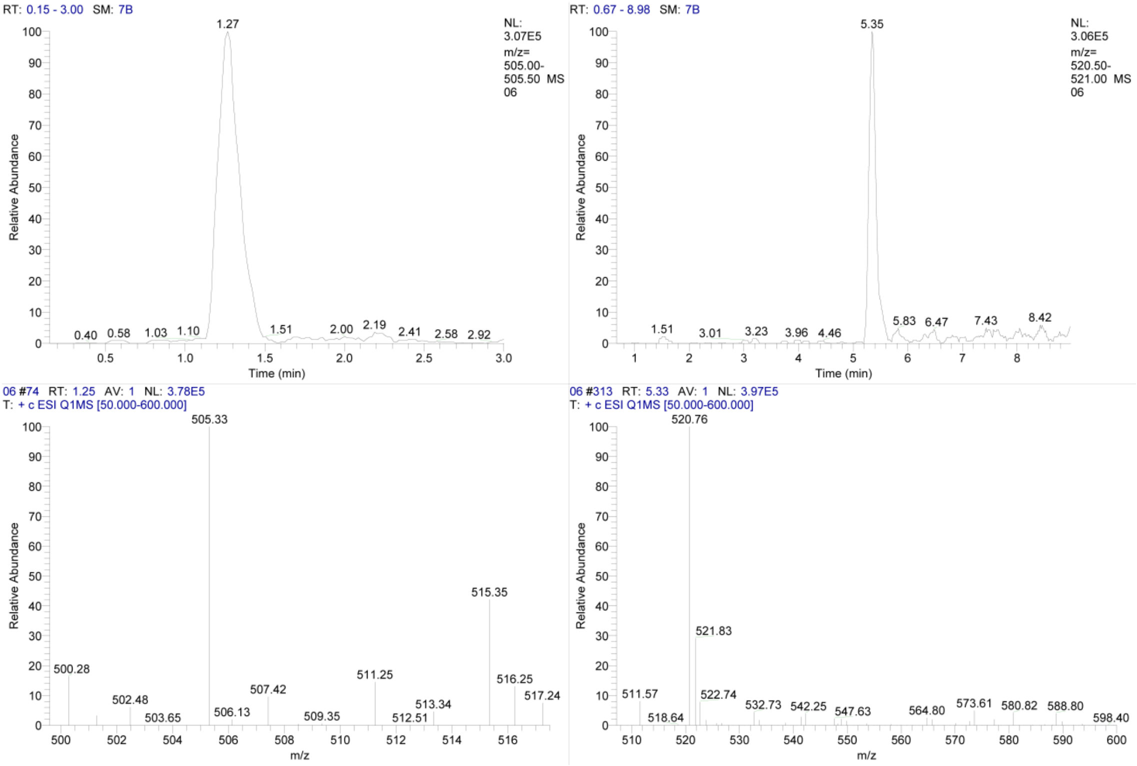

RT: 0.15 - 3.00 SM: 7B
NL: 3.07E5 m/z= 505.00-505.50 MS 06
1.27
Relative Abundance
Time (min)
RT: 0.67 - 8.98 SM: 7B
NL: 3.06E5 m/z= 520.50-521.00 MS 06
5.35
06 #74 RT: 1.25 AV: 1 NL: 3.78E5
T: + c ESI Q1MS [50.000-600.000]
505.33
515.35
m/z
06 #313 RT: 5.33 AV: 1 NL: 3.97E5
T: + c ESI Q1MS [50.000-600.000]
520.76
521.83

Figure S17. Numbers of the atoms in SMX

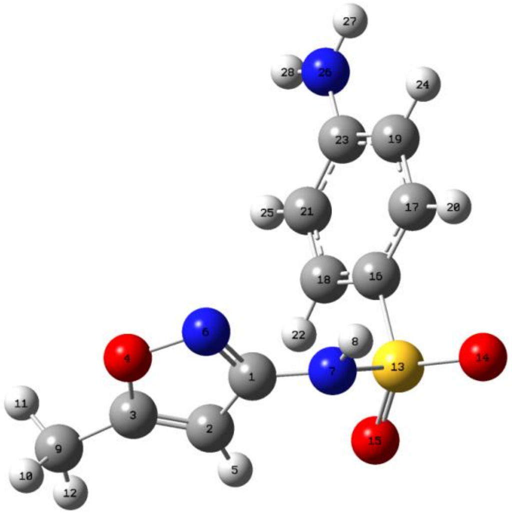

Reference

[1] S. Zhu, X. Li, J. Kang, X. Duan, S. Wang, Persulfate activation on crystallographic manganese oxides: Mechanism of singlet oxygen evolution for nonradical selective degradation of aqueous contaminants, Environ Sci Technol 53 (2019) 307-315.
[2] J. Wang, S. Wang, Reactive species in advanced oxidation processes: Formation, identification and reaction mechanism, Chemical Engineering Journal 401 (2020).
